# Mutation in VPS35 associated with Parkinson’s disease impairs WASH complex association and inhibits autophagy

**DOI:** 10.1038/ncomms4828

**Published:** 2014-05-13

**Authors:** Eszter Zavodszky, Matthew N.J. Seaman, Kevin Moreau, Maria Jimenez-Sanchez, Sophia Y. Breusegem, Michael E. Harbour, David C. Rubinsztein

**Affiliations:** 1 Department of Medical Genetics, Cambridge Institute for Medical Research, Cambridge Biomedical Campus, University of Cambridge, Wellcome Trust/MRC Building, Hills Road, Cambridge CB2 0XY, UK; 2 Department of Clinical Biochemistry, Cambridge Institute for Medical Research, University of Cambridge, Wellcome Trust/MRC Building, Addenbrooke’s Hospital, Cambridge CB2 0XY, UK; 3 These authors contributed equally to this work

## Abstract

Endosomal protein sorting controls the localization of many physiologically important proteins and is linked to several neurodegenerative diseases. VPS35 is a component of the retromer complex, which mediates endosome-to-Golgi retrieval of membrane proteins such as the cation-independent mannose 6-phosphate receptor. Furthermore, retromer is also required for the endosomal recruitment of the actin nucleation promoting WASH complex. The VPS35 D620N mutation causes a rare form of autosomal-dominant Parkinson’s disease (PD). Here we show that this mutant associates poorly with the WASH complex and impairs WASH recruitment to endosomes. Autophagy is impaired in cells expressing PD-mutant VPS35 or lacking WASH. The autophagy defects can be explained, at least in part, by abnormal trafficking of the autophagy protein ATG9A. Thus, the PD-causing D620N mutation in VPS35 restricts WASH complex recruitment to endosomes, and reveals a novel role for the WASH complex in autophagosome formation.

## Abstract (web summary)

Parkinson’s disease can be caused by a rare mutation in the protein VPS35, but the mechanism responsible for this is largely unknown. Here, Zavodszky *et al.* show that this mutation leads to defects in the recruitment of endosomal protein sorting machinery and consequent inhibition of autophagy in cells.

## Graphical abstract

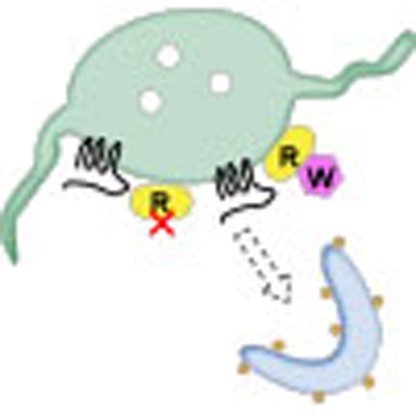

The retromer complex is a conserved membrane-associated protein complex that functions in the endosome-to-Golgi retrieval pathway. Retromer consists of a cargo-selective complex (CSC) comprising VPS35, VPS26 and VPS29, along with a sorting nexin dimer consisting of SNX1 or SNX2 with SNX5 or SNX6. Many membrane proteins (often referred to as ‘cargo’) depend on retromer for their respective localization^1^. A well-studied cargo protein for retromer-mediated endosome-to-Golgi retrieval is the cation-independent mannose 6-phosphate receptor (CIMPR) that operates as a lysosomal hydrolase receptor, sorting acid hydrolases for exit from the trans-Golgi network (TGN) before returning to the Golgi via the endosome-to-Golgi retrieval pathway^2^,^3^.

Recently, it has been shown that retromer function is important for more than endosome-to-Golgi retrieval. The retromer complex is responsible for the endosomal recruitment of the WASH complex^4^, a protein complex that, together with Arp2/3, mediates actin patch formation on endosomes to facilitate protein sorting^5^,^6^. WASH complex activity is especially important for the endosome-to-cell surface recycling of certain membrane proteins, including the β2-adrenergic receptor, α5β1 integrin, the glucose transporter GLUT-1, and the T-cell receptor in T-cells^7^,^8^,^9^, reviewed previously^10^. Cargo proteins that require the activity of the WASH complex for their trafficking are therefore indirectly dependent on retromer, even if retromer does not participate directly in their sorting. The recruitment of the WASH complex to endosomes is mediated by interactions between the extended tail of the FAM21 protein of the WASH complex and VPS35 in the retromer CSC^11^,^12^,^13^. In addition, a protein called FKBP15 (also known as FKBP133 and WAFL), which has been implicated in nerve growth-cone collapse, interacts with both the FAM21 tail and VPS35, although its function with the WASH and retromer complexes is unclear^4^,^11^,^14^.

Impaired endosomal protein sorting underlies many neurological diseases. One component of the WASH complex, strumpellin, is mutated in hereditary spastic paraplegia^15^,^16^. Mutation of another component of the WASH complex, KIAA1033 (also known as SWIP), results in autosomal recessive intellectual disability^17^. In addition, recent reports have revealed that Parkinson’s disease (PD) can be caused by a rare autosomal-dominant *VPS35* mutation that results in an aspartate to an asparagine substitution at residue 620 (D620N)^18^,^19^. The mechanism through which the D620N mutation exerts its effects is presently unknown.

Macroautophagy (hereafter referred to as autophagy) is a degradative pathway by which the breakdown of cellular proteins releases amino acids necessary for survival, particularly under starvation or stress conditions^20^. Autophagy also removes damaged mitochondria and certain intracellular pathogens, such as *Salmonella*^21^,^22^. Moreover, autophagy is of profound importance in the clearance of intracytoplasmic aggregate-prone proteins, such as those formed by the polyglutamine-expanded repeats in huntingtin, the Huntington’s disease-causing protein, as well as α-synuclein, the major component of PD-associated Lewy bodies^23^,^24^,^25^. In fact, autophagy appears to be compromised in the brains of PD patients^26^, and excess α-synuclein inhibits autophagosome formation^27^. The autophagic pathway is especially sensitive to perturbations of endosomal protein sorting, and thus makes a plausible downstream target of defects associated with retromer-mediated sorting. In fact, depletion of yeast retromer components impairs autophagic activity, although results in mammalian cells are at present unclear^28^.

Here we show that the PD-causing mutation in VPS35 destabilizes the retromer–WASH complex interaction, leading to reduced endosomal localization of the WASH complex. We further show that the WASH complex is necessary for autophagosome formation and that cells expressing the PD-causing *VPS35* allele exhibit defects in autophagy, as well as in the trafficking of the multipass transmembrane autophagy protein ATG9A. This provides important mechanistic insights into the pathology of the PD-causing *VPS35* mutation and demonstrates a novel role for the WASH complex in autophagy.

## Results

### VPS35 D620N exhibits impaired binding to the WASH complex

**Figure 1:**
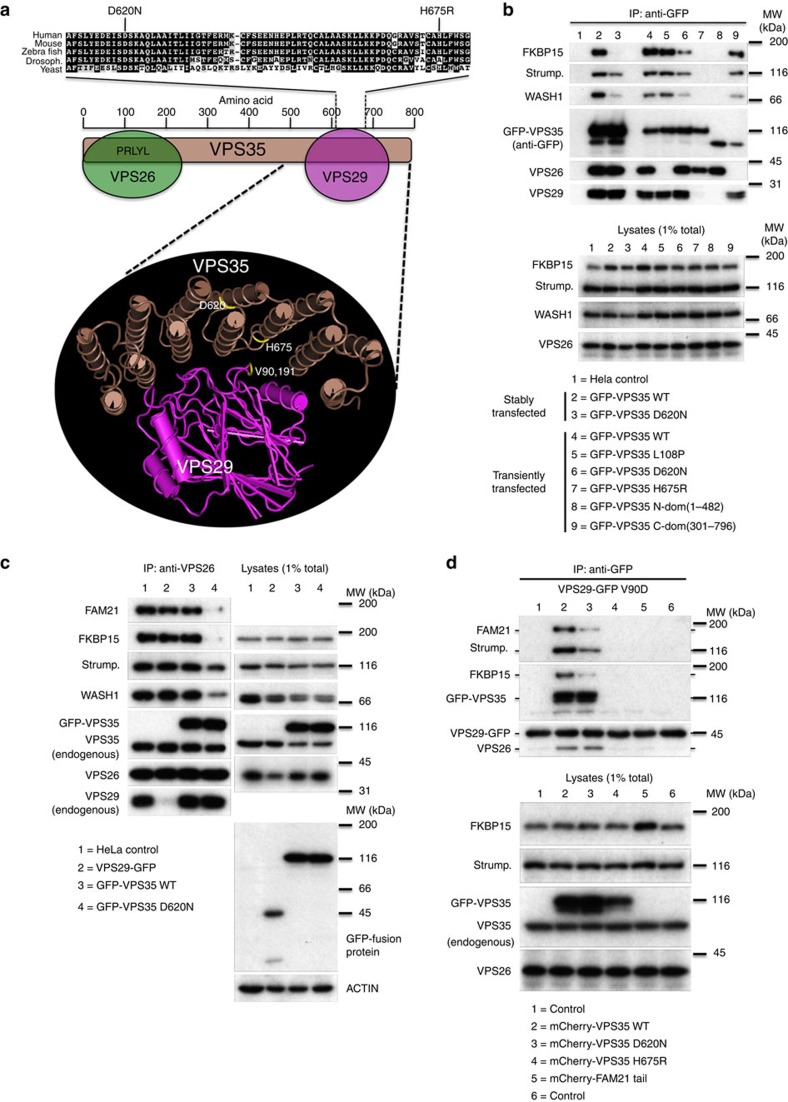
Effect of the D620N mutation on the assembly and protein–protein interactions of the retromer CSC. (**a**) Schematic diagram showing an alignment of the human VPS35 sequence (residues 610–680) with homologues from other well-studied eukaryotes. The D620N mutation is highlighted along with the H675R mutation that blocks binding to VPS29. The position of the D620N mutation of VPS35 is shown on the three-dimensional structure along with the H675R mutation and the V90 and I91 residues of VPS29 that mediate binding to VPS35 (PDB ID. 2R17). (**b**) Cells stably expressing WT or D620N GFP-VPS35 or otherwise transiently transfected with a panel of GFP-VPS35 constructs were lysed and the respective GFP-tagged protein recovered by immunoprecipitation (IP). (**c**) Untransfected HeLa cells or cells stably expressing VPS29-GFP, GFP-VPS35 wild-type or GFP-VPS35 D620N were lysed and the lysates incubated with anti-VPS26 to IP the retromer CSC. (**d**) Cells stably expressing the VPS29-GFP V90D mutant that cannot assemble with VPS35 were transiently transfected with mCherry-tagged WT VPS35, D620N and H675R constructs, along with an additional control of mCherry-FAM21 tail. Cells were lysed 48 h post transfection, and VPS29-GFP V90D and associated proteins were recovered by anti-GFP native IP. Blots shown are representative of experiments replicated at least twice.

Figure 1a shows human VPS35 residues 610–680 aligned with VPS35 homologues from other well-studied eukaryotes. The D620N mutation falls within the horseshoe-shaped region that comprises many helical repeats and folds around VPS29 (ref. 29). We also highlight the recently identified H675R VPS35 mutation that abolishes binding to VPS29, an interaction that is required for the subsequent association of VPS35 and the WASH complex^12^. As the D620N mutation lies within the VPS29-binding region, we tested whether it affected the interaction between VPS35 and VPS29, as well as with other retromer-interacting proteins.

In Fig. 1b, cells stably expressing green fluorescent protein (GFP)-tagged wild-type (WT) VPS35 or VPS35 D620N were lysed along with untransfected HeLa cells and incubated with anti-GFP antisera (lanes 1–3). Similarly, HeLa cells were transiently transfected with a panel of GFP-VPS35 constructs, and lysates were incubated with anti-GFP to recover the expressed protein (lanes 4–9). Both stably and transiently transfected WT VPS35 co-immunoprecipitated the VPS26 and VPS29 retromer CSC subunits, along with strumpellin and WASH1 of the WASH complex as well as FKBP15 (lanes 2 and 4). The D620N mutant associated normally with both VPS26 and VPS29 but showed reduced associations with FKBP15 and the WASH complex proteins (compare lanes 2 with 3 and 4 with 6). The effect of the D620N mutant was not, however, as pronounced as that of the H675R mutant, which cannot bind VPS29 and completely fails to associate with the WASH complex (compare lanes 6 and 7). The VPS35 L108P mutation within the conserved PRLYL motif (lane 5) blocked binding to VPS26 (refs 30, 31, 32) but did not affect interactions with either VPS29 or the WASH complex^12^. Two truncation constructs of VPS35 confirmed that binding of the WASH complex and FKBP15 to VPS35 occurs independently of VPS26 but requires the carboxy-terminal region of VPS35 and proper interaction with VPS29.

We next used antisera against VPS26 to immunoprecipitate the retromer CSC and determine the effect the D620N mutation on the association of the CSC with the WASH complex. Components of the WASH complex (FAM21, strumpellin and WASH1) were readily detected in association with the retromer CSC in HeLa cells, as well as cells stably expressing WT VPS29 or WT VPS35, but not in cells expressing VPS35 D620N (Fig. 1c). The reduced level of endogenous VPS29 that co-precipitated using the anti-VPS26 antibody in the VPS29-GFP lysate was due to the expression of the GFP-tagged VPS29 that competes for binding to VPS35 displacing the endogenous VPS29—an effect not observed when a mutant of VPS29 (V90D) that interacts weakly with VPS35 (ref. 33) is expressed instead (Supplementary Fig. 1a). Besides competing with endogenous VPS29, the expression of VPS29-GFP does not affect the interactions of the retromer CSC.

To test if the VPS35 D620N mutation affects its association with VPS29 using another approach, we employed cells stably expressing VPS29 V90D. We have previously reported that transient overexpression of WT VPS35 can overcome the VPS29 V90D mutation and enable the mutant VPS29 protein to co-immunoprecipitate both VPS26 and the WASH complex^12^. The VPS35 H675R mutant, however, fails to rescue VPS29 V90D^12^. Therefore, in Fig. 1d, cells stably expressing VPS29-GFP V90D were transiently transfected with mCherry-tagged WT VPS35, D620N or H675R as well as mCherry-FAM21 tail as an additional control. In lane 2, transient expression of WT VPS35 enabled VPS29 V90D to co-immunoprecipitate VPS26, FKBP15 as well as the WASH complex proteins FAM21 and strumpellin. The H675R mutant (lane 4) cannot interact with VPS29 and therefore could not rescue the VPS29 V90D mutant. The D620N mutant (lane 3) interacted with the VPS29 V90D mutant and promoted an association with VPS26 that was indistinguishable from WT VPS35, but markedly less FAM21 and strumpellin proteins (that is, WASH complex) as well as FKBP15 associated with the VPS29-GFP V90D protein. The expression of the mCherry-tagged FAM21 tail did not affect the interactions of VPS29 (lane 5).

**Figure 2:**
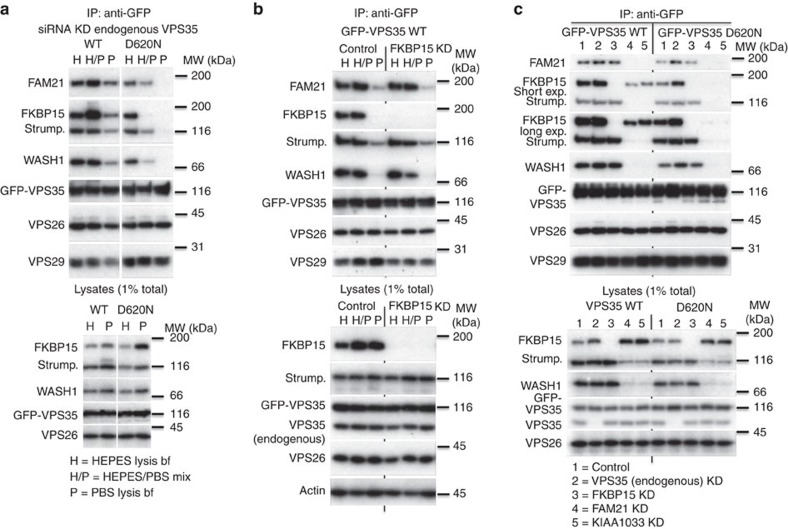
The D620N mutation destabilizes the retromer–WASH complex association. (**a**) Cells expressing either WT GFP-VPS35 or the D620N mutant were treated with siRNA to abolish expression of endogenous VPS35. Each dish of cells was lysed in either HEPES lysis buffer (H) or PBS lysis buffer (P). Following centrifugation, equal portions of each lysate were combined to generate a mixed lysate (H/P). Each lysate was incubated with anti-GFP to recover the respective GFP-tagged VPS35 protein. (**b**) Similar to (**a**), but only cells expressing GFP-VPS35 wild type were analysed, either mock treated or treated with siRNA to silence FKBP15 expression. (**c**) Cells stably expressing WT or D620N GFP-VPS35 were treated with indicated siRNAs, lysed and immunoprecipitated as in (**a**).

The data presented in Fig. 1 suggest that the VPS35 D620N mutation does not affect VPS29 binding or CSC assembly, but reduces interaction with the WASH complex and FKBP15. To further explore these effects, cells stably expressing WT VPS35 or the D620N mutant were lysed using buffers of differing stringencies. The HEPES-based buffer has lower stringency, while the phosphate-buffered saline (PBS) lysis buffer has a higher stringency, as it contains more salt and detergent. Following lysis, samples of equal volume of the HEPES and PBS lysates were mixed to produce a lysate of intermediate stringency. To ensure that the GFP-tagged constructs could associate with FKBP15 or the WASH complex without competition, small interfering RNA (siRNA) was used to silence the expression of endogenous VPS35. The GFP-tagged murine VPS35 constructs are resistant to the siRNA targeting endogenous VPS35. As before, WT VPS35 co-immunoprecipitated WASH complex components WASH1, FAM21, strumpellin, as well as FKBP15, with some reduction in the levels of WASH complex proteins and FKBP15 in the higher stringency PBS buffer (Fig. 2a). The D620N mutant, however, exhibited a marked reduction in both WASH complex and FKBP15 binding in the intermediate stringency buffer (H/P) and no association in the higher stringency PBS buffer (P). Levels of VPS26 and VPS29 did not change significantly, indicating that there was no destabilization of the retromer complex due to either the D620N mutation or the different lysis buffers.

The sensitivity of the retromer–WASH complex association to different lysis buffer conditions was confirmed in Supplementary Fig. 1b, where cells stably expressing either GFP-tagged VPS29 or the FAM21 tail were lysed with either HEPES, PBS or the mixed lysis buffer prior to immunoprecipitation (IP) with anti-GFP. In this experiment, interactions between retromer and both the WASH complex and FKBP15 are lost with increasing lysis buffer stringency, but the interactions that underpin assembly of the retromer CSC are retained.

### VPS35 binding to WASH complex is independent of FKBP15

The D620N mutation affects binding of VPS35 to the WASH complex and almost abolishes association with FKBP15. To determine if loss of the VPS35-FKBP15 interaction is destabilizing the retromer–WASH complex interaction, cells expressing WT GFP-VPS35 were treated with siRNA to silence FKBP15 expression. Lysates were then generated using the buffers of differing stringency and GFP-tagged VPS35 recovered by IP. In Fig. 2b, there was no pronounced change in the association of the WASH complex with WT GFP-VPS35 after FKBP15 depletion, indicating that the D620N-mediated loss of FKBP15 interaction with VPS35 does not cause the defective WASH complex association with the D620N VPS35 mutant.

To further delineate the defects associated with the D620N mutant, the cells stably expressing GFP-tagged WT or D620N VPS35 were treated with siRNA to silence the expression of either endogenous VPS35, FKBP15 or the FAM21 or KIAA1033 components of the WASH complex. It has been shown previously that knockdown of one WASH complex protein affects the stability and levels of the others^11^,^34^. Following knockdown, lysates were incubated with anti-GFP to recover VPS35 (Fig. 2c). For this experiment, twice the amount of material used in previous experiments was analysed using the HEPES-based buffer to create conditions that can be considered the most permissive for retaining retromer–WASH complex interactions. WT VPS35 interacted with the WASH complex in the absence of FKBP15, as demonstrated by the presence of strumpellin and WASH1 in lane 3. Similarly, FKBP15 co-immunoprecipitated with WT VPS35 after siRNA knockdown of FAM21 or KIAA1033, although at reduced levels (see lanes 4 and 5). In contrast, the D620N mutant failed to co-immunoprecipitate FKBP15 in cells where the WASH complex was silenced but retained an interaction with WASH complex proteins when FKBP15 expression was abolished. Thus, binding of the WASH complex to retromer appears to occur largely independently of FKBP15. This is consistent with co-IP of WASH components by GFP-tagged VPS29 after knockdown of FKBP15, as seen in Supplementary Fig. 1c and in previous work^4^.

Although FKBP15 is not strictly necessary for the retromer–WASH complex association, increasing levels of FKBP15 by transient transfection can enhance the retromer–WASH complex interaction in the higher stringency PBS lysis buffer for WT VPS35 but not for the D620N mutant (Supplementary Fig. 1d).

### VPS35 D620N impairs WASH complex recruitment to endosomes

**Figure 3:**
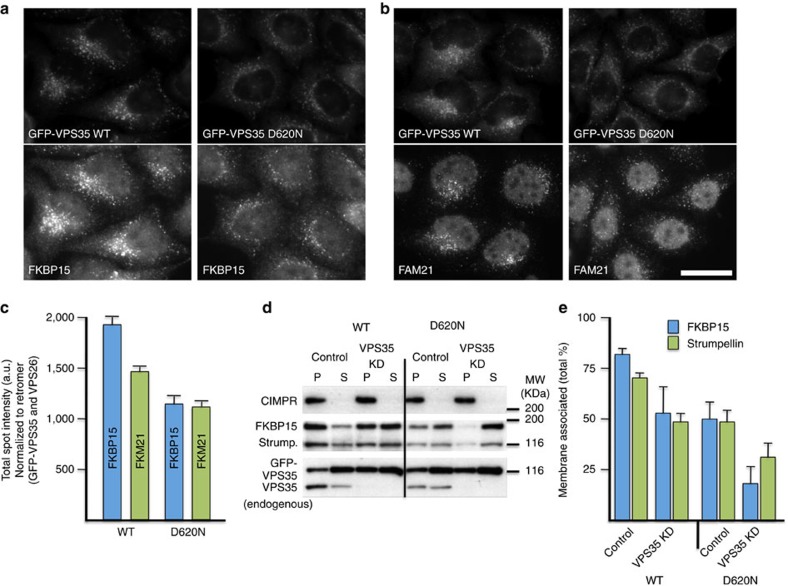
The D620N mutation leads to reduced endosomal association of FKBP15 and the WASH complex. (**a**,**b**) Cells expressing either WT or D620N GFP-VPS35 were treated with siRNA to silence expression of endogenous VPS35, fixed and labelled for GFP and FKBP15 (**a**) or FAM21 (**b**). Cells were imaged by fluorescence microscopy. Scale bar, 20 μm. (**c**) Cells stably expressing WT or D620N GFP-tagged VPS35 were treated with siRNA to abolish expression of endogenous VPS35 and subsequently fixed and labelled for GFP and either VPS26, FKBP15 or FAM21. The cells were imaged using Cellomics automated fluorescence microscopy. Approximately 250 cells per well in four wells were analysed for each cell line. The data for FKBP15 and FAM21 spot intensity were normalized to GFP-VPS35 and VPS26 signals. *P*<0.0002 for both FKBP15 and FAM21 spot intensity in D620N compared with WT. Error bars indicate s.d. (**d**) Cells stably expressing either WT GFP-VPS35 or the D620N mutant were treated with siRNA to silence the expression of endogenous VPS35. Cells were permeabilized by flash freezing followed by rapid thawing and centrifuged to separate supernatant (S) and membrane pellet (P) fractions. (**e**) The graph shows the percentage of membrane-associated FKBP15 and strumpellin and is the mean of three experiments. The error bars indicate s.e.m. A representative blot of three experiments is shown, indicating the efficacy of the knockdown of endogenous VPS35 and also further showing that membrane proteins such as the CIMPR are detected only in the pellet fraction.

Retromer mediates the endosomal recruitment of both the WASH complex and FKBP15 (ref. 4). VPS35 recruits the WASH complex through interactions with the FAM21 tail^11^,^12^,^13^. To test the effect of the D620N mutation on the localization of FKBP15 and the WASH complex, cells stably expressing either WT GFP-VPS35 or the D620N mutant were treated with siRNA to silence endogenous VPS35 expression. After fixation, the cells were double-labelled with a monoclonal anti-GFP antibody and antisera against FKBP15 or FAM21 (Fig. 3a,b). Quantification of the fluorescence intensities indicates that the D620N-expressing cells have reduced endosomally localized FKBP15 and FAM21 compared with the WT cells, compatible with decreased endosomal recruitment (Fig. 3c).

To more rigorously examine the effect of the D620N mutation on the membrane association of FKBP15 and the WASH complex, we separated cytoplasmic proteins in the supernatant (S) from membrane and membrane-associated proteins in the pellet (P) fraction by centrifugation. Cells expressing the D620N mutant had less membrane-associated FKBP15 and strumpellin both in the presence of endogenous VPS35 and most significantly when endogenous VPS35 was silenced (Fig. 3d,e). The cells expressing WT VPS35 showed some reduction in the membrane association of FKBP15 and strumpellin when endogenous VPS35 was silenced, possibly due to minor inhibitory effects of the GFP moiety added to the amino terminus of VPS35. These data, taken together, demonstrate that the D620N mutation results in a reduction of the membrane association of FKBP15 and the WASH complex.

### VPS35 D620N expression mimics WASH-associated phenotypes

Thus far, we have reported an impaired association of the D620N mutant for the WASH complex and FKBP15 but the functional significance has not been elucidated. Previous experiments have shown that siRNA knockdown of FKBP15 does not impede endosome-to-Golgi retrieval of a CD8-CIMPR chimera reporter protein^4^. Similarly, D620N-expressing cells exhibited apparently normal VPS26, SNX1, CIMPR, and LAMP1 localization (Supplementary Fig. 2a–c).

We observed a cell spreading defect in cells expressing VPS35 D620N, compared with their WT counterparts (Supplementary Fig. 3a), which we confirmed in independent clones. Cell spreading defects also occur in cells overexpressing the FAM21 tail, which competes with the endogenous WASH complex for binding to retromer^11^ or WASH1 knockdown cells^9^. Thus, the cell spreading defect in the VPS35 D620N cells is consistent with the reduced affinity of the mutant VPS35 for the WASH complex, resulting in WASH complex mislocalization. While CIMPR and retromer exhibited normal localization in the D620N-mutant cells, we observed altered localization of GLUT-1, membrane protein that requires the WASH complex for its steady-state localization to the cell surface^7^,^35^. The cells expressing the VPS35 D620N mutant displayed reduced cell surface staining of GLUT-1 and increased intracellular staining (Supplementary Fig. 3b). As the input lysates in Figs 1, 2 showed that WASH1 levels remain essentially the same in both the VPS35 WT and D620N cell lines, the WASH-related phenotypes observed in D620N cells are not due to changes in the steady-state level of WASH1 but likely due to WASH complex mislocalization.

### VPS35 D620N impairs autophagy

**Figure 4:**
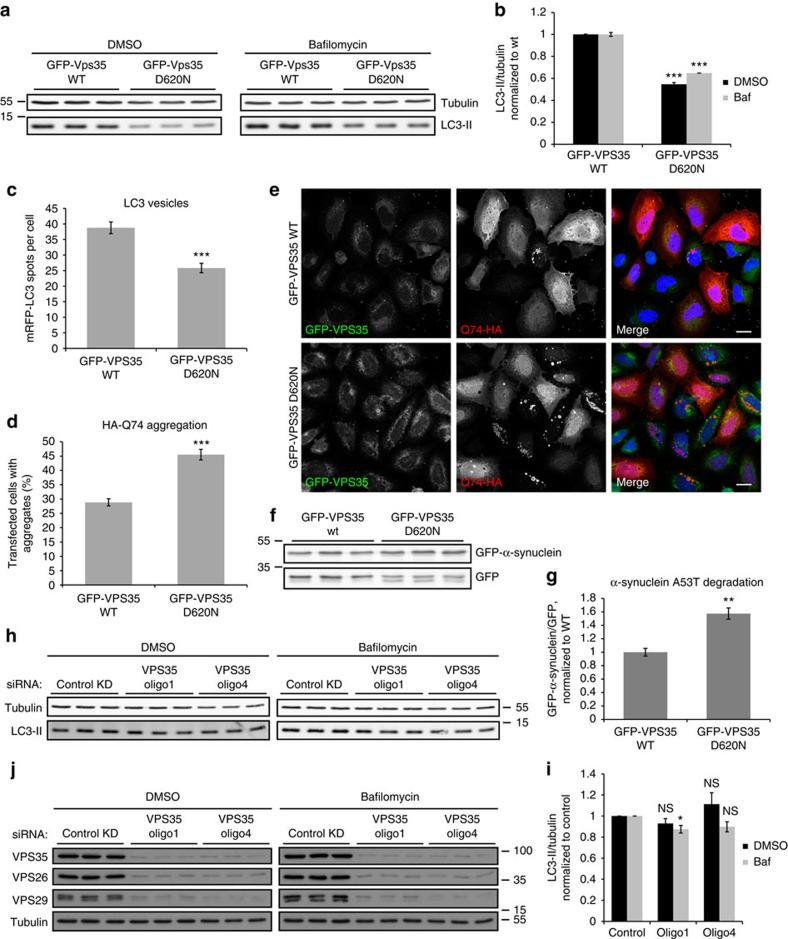
VPS35 D620N impairs autophagy, while VPS35 knockdown has only modest effects. (**a**) HeLa cells stably expressing GFP-VPS35 WT and D620N were treated with bafilomycin A1 or DMSO vehicle control. Endogenous LC3-II and tubulin levels were examined by western blot. A representative experiment of six experiments is shown. (**b**) Quantification of the representative experiment in triplicate shown in **a**, in which endogenous LC3-II levels are normalized to tubulin and expressed as a ratio of levels in WT. ***P=7.86 × 10^−6^ (DMSO) and 3.81 × 10^−5^ (Baf) by 2-tailed Student’s *t*-test. (**c**) HeLa cells stably expressing GFP-VPS35 WT and D620N were transfected with mRFP-LC3. The number of LC3 vesicles was quantified by Cellomics automated fluorescence microscopy. A representative experiment of three independent experiments is shown, with 344 (WT) and 401 (D620N) cells analysed. ***P<0.0001 by 2-tailed Student’s *t*-test. (**d**) GFP-VPS35 WT and D620N-expressing cells were transfected with HA-Q74 and immunostained for HA. The percentage of transfected cells with aggregates was counted by a blinded experimenter. The quantification shows the mean of three experiments in triplicate with minimum 200 cells per replicate. ***P=0.00086 by 1-tailed Student’s *t*-test. (**e**) Confocal images representative of the experiment described in **d**. Scale bar, 20 μm. (**f**) Cells stably expressing WT and D620N GFP-VPS35 were transfected with GFP-α-synuclein A53T and GFP for 48 h and analyzed by western blotting. (**g**) Quantification of the representative experiment in triplicate in **f**, in which the level of α-synuclein was expressed as a ratio to GFP. A representative experiment of two independent experiments is shown. **P=0.0047 by 2-tailed Student’s *t*-test. (**h**) VPS35 was knocked down with two individual siRNA nucleotides in HeLa cells, and cells were treated with bafilomycin A1 and lysed as in **a**. A representative experiment is shown in triplicate. (**i**) Quantification of three independent experiments in triplicate. *P=0.026; other results non-significant by 2-tailed Student’s *t*-test. (**j**) Protein levels of CSC, including VPS35, VPS26 and VPS29), were assessed upon VPS35 knockdown, confirming previous results that knockdown of one component destabilizes the CSC. All error bars indicate s.e.m.

Proper endosomal function is important for autophagy, prompting us to test if the VPS35 D620N mutation affects this process. Autophagy can be measured by examining levels of LC3-II, the only protein known to associate specifically with autophagosomes, as its levels correlate with autophagosome numbers. Because changes in LC3-II levels and autophagosome numbers may be due to altered autophagosome formation or degradation, one can uncouple these processes using drugs like bafilomycin A1, which blocks autophagosome degradation^36^,^37^, thus allowing assessment of changes in formation alone. Interestingly, the stable GFP-VPS35 D620N cell lines showed lower LC3-II levels than their WT counterparts both with and without bafilomycin, indicating defective autophagosome formation (Fig. 4a,b). Furthermore, Supplementary Fig. 4a,b replicates this result in independent clones of GFP-VPS35 WT and D620N cells. In addition, knocking down endogenous VPS35 in both sets of clones stably expressing GFP-VPS35 wild type and D620N did not appear to alter the levels of LC3-II, as the difference between WT and mutant cells remained pronounced, regardless of the presence of endogenous VPS35 (Supplementary Fig. 4c–e). Upon transient transfection of a fluorescent LC3 construct, we observed fewer punctate LC3 structures in cells expressing GFP-VPS35 D620N, compared with wild-type, indicating fewer autophagosomes (Fig. 4c).

To confirm a functional defect in autophagy, we transfected the cell lines with a hemagglutinin (HA)-tagged aggregation-prone model autophagy substrate, huntingtin exon 1 containing 74 glutamine repeats. The percentage of transfected cells with huntingtin aggregates correlates directly with huntingtin abundance, which is, in turn, autophagy dependent^23^. The D620N-mutant cells consistently had a higher percentage of transfected cells containing aggregates, consistent with impaired autophagosome formation and autophagic clearance (Fig. 4d,e). Furthermore, cells expressing VPS35 D620N showed increased levels of another autophagy substrate, PD-variant α-synuclein A53T, relative to GFP (Fig. 4f,g).

VPS35 depletion by RNA interference only resulted in modest changes in LC3-II levels, as seen in Fig. 4h–j, consistent with recent data using VPS29 depletion^38^. This is likely because loss of VPS35 destabilizes the entire retromer CSC and has wide-ranging effects on multiple pathways, including defects in CIMPR retrieval that impact cathepsin D trafficking and lysosome biogenesis. Consequently, VPS35 depletion will have pleiotropic consequences on endosomal protein sorting, resulting in the possible masking of the autophagosome formation defect by other compensatory effects.

### Perturbation of WASH complex binding disrupts autophagy

**Figure 5:**
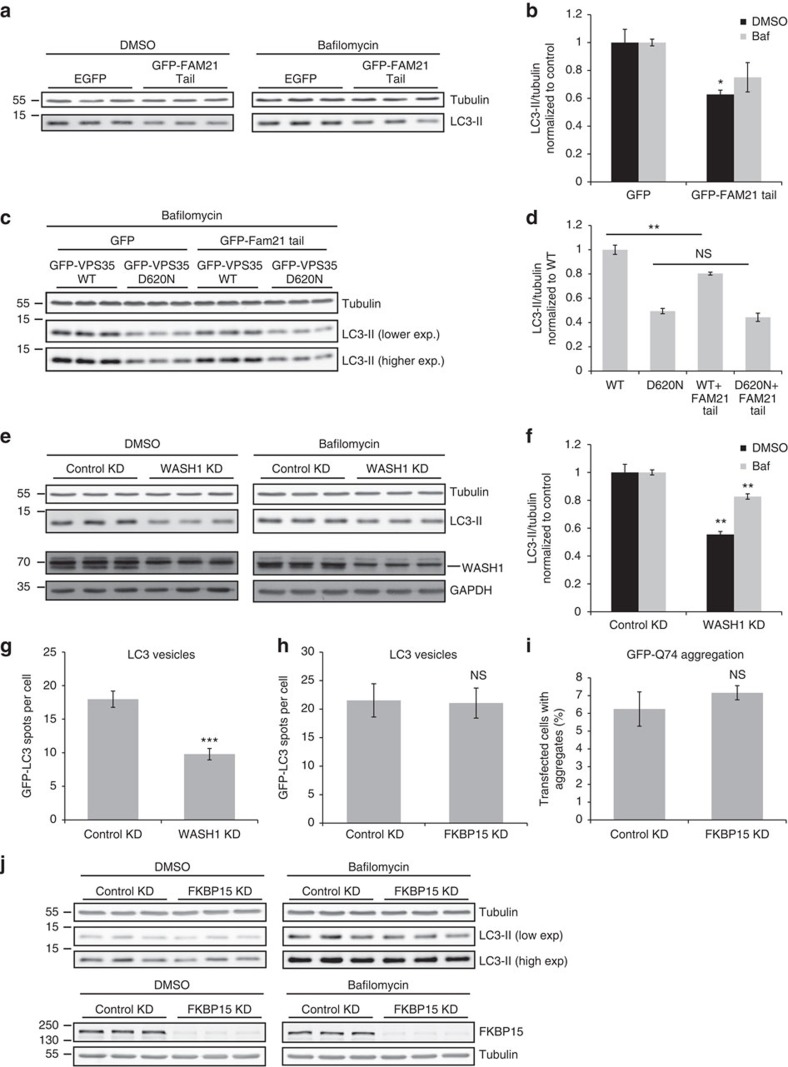
WASH complex binding to retromer is necessary for autophagy. (**a**) HeLa cells were transfected with pEGFP vector or GFP-FAM21 tail for 48 h and subsequently treated with bafilomycin A1 as in Fig. 4. Endogenous LC3-II and tubulin levels were assessed by western blot. (**b**) Quantification of the representative experiment in triplicate shown in **a**, of two independent experiments. *P=0.02 (DMSO) and 0.08 (Baf) by 2-tailed Student’s *t*-test. (**c**) HeLa cells stably expressing GFP-VPS35 WT and D620N were transfected with pEGFP vector or GFP-FAM21, and subsequently treated with bafilomycin A1, lysed and subjected to western blot as in **a**. (**d**) Quantification of the representative experiment in triplicate shown in **c**, of two independent experiments. **P=0.008 by 2-tailed Student’s *t*-test. (**e**) Cells were depleted of WASH1, treated with bafilomycin A1 and examined for endogenous LC3-II, tubulin, WASH1 and GAPDH levels. A representative experiment of six independent experiments is shown. (**f**) Quantification of the representative experiment shown in **e**. **P=0.002 (DMSO) and *P*=0.0028 (Baf) by 2-tailed Student’s *t*-test. (**g**) HeLa cells depleted of WASH1 by siRNA were transfected with GFP-LC3 for 24 h. The number of LC3 vesicles was quantified by Cellomics automated fluorescence microscopy. A representative experiment of two independent experiments is shown, with 757 (control) and 695 (knockdown) cells analysed. ***P<0.0001 by 2-tailed Student’s *t*-test. (**h**) HeLa cells depleted of FKBP15 were transfected with GFP-LC3 and vesicles were quantified as in **g**. A representative experiment of two independent experiments in triplicate is shown, with 579 (control) and 815 (knockdown) cells analysed in total. (**i**) HeLa cells depleted of FKBP15 were subsequently transfected with a GFP-Q74 construct for 24 h and fixed in PFA. The percentage of transfected cells with aggregates was counted by a blinded experimenter. The quantification shows the mean of two experiments, each in triplicate with min 220 cells counted per replicate. (**j**) FKBP15 was depleted in HeLa cells as in **e**. A representative blot from three independent experiments in triplicate is shown. Error bars indicate s.e.m.

As VPS35 D620N exhibits decreased affinity for the WASH complex, we mimicked this reduced binding by overexpressing the FAM21 tail, which displaces the endogenous WASH complex from retromer^11^,^12^,^13^. FAM21 tail overexpression reproducibly reduced LC3-II levels both with and without bafilomycin (Fig. 5a,b). The effect was not as pronounced as that in the stably expressing D620N cells, as FAM21 tail was transiently transfected and hence not present in every cell.

In an epistasis experiment where the FAM21 tail was expressed in the VPS35 stable cell lines, we observed that overexpression of the FAM21 tail decreased autophagosome formation (as indicated by reduced LC3-II levels under bafilomycin-treated conditions) in WT cells but not in D620N-mutant cells (Fig. 5c,d). In the mutant line, WASH complex binding to retromer is already impaired and thus overexpression of the FAM21 tail has no additive influence, suggesting that the observed effects on autophagosome formation are indeed due to the decreased association between VPS35 and the WASH complex. The importance of WASH complex activity for autophagosome formation was underscored by finding that siRNA knockdown of WASH1 reduced LC3-II levels with and without bafilomycin (Fig. 5e,f). In addition, depletion of WASH1 leads to fewer GFP-LC3 vesicles, again pointing to the impaired autophagosome formation (Fig. 5g).

While the VPS35 D620N mutation hinders retromer binding not only to the WASH complex but also to FKBP15, knockdown of FKBP15 had a much less robust effect on autophagy than WASH1 depletion, as evidenced by only modest, non-statistically significant effects on endogenous LC3-II levels, no difference in GFP-LC3 vesicle numbers, and no difference in the percentage of cells with mutant huntingtin aggregates (Fig. 5h–j). This is consistent with the hypothesis that FKBP15 is not an essential part of the complex and that that the WASH complex can still bind to retromer in the absence of FKBP15 (Fig. 2b,c).

### VPS35 D620N and WASH depletion affect trafficking of ATG9A

The autophagy pathway requires multiple autophagy-related genes, including ATG9A, a multipass transmembrane protein. Under basal conditions, mammalian ATG9A localizes to the TGN and recycling endosomes, and redistributes to a more peripheral pool after autophagy induction by nutrient deprivation (reviewed by Zavodszky *et al.*^39^).

**Figure 6:**
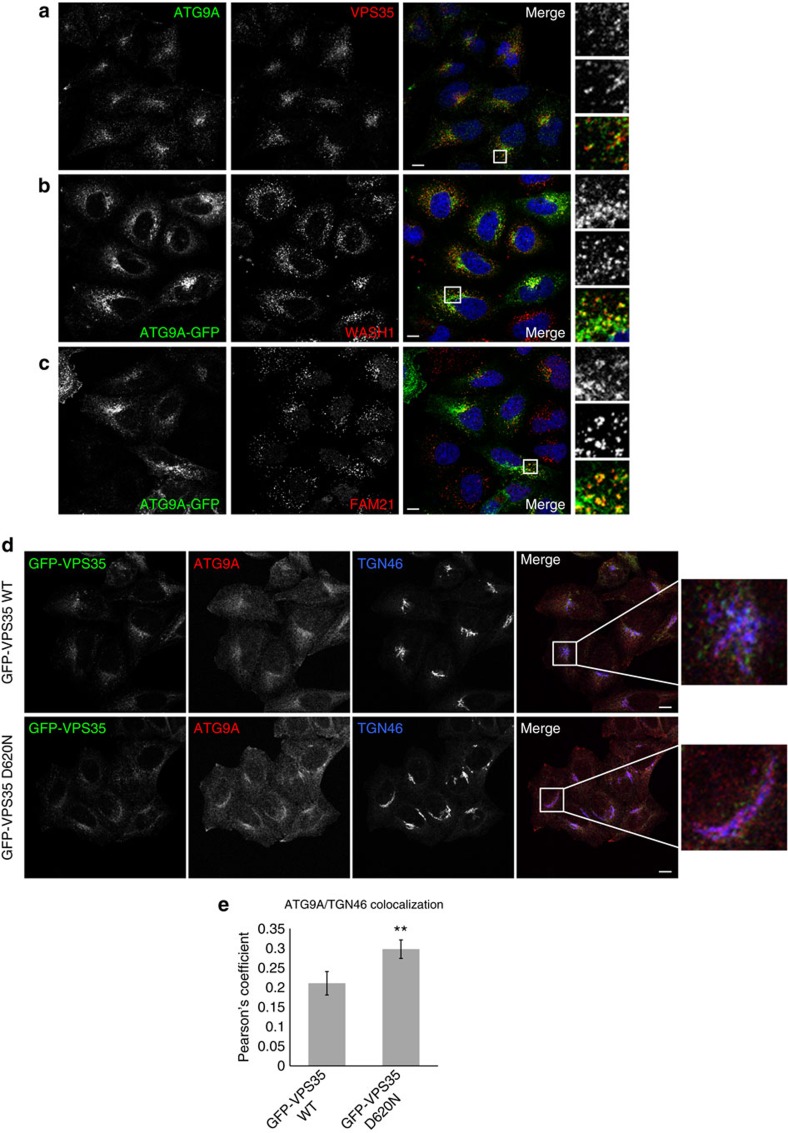
VPS35 D620N affects trafficking and localization of ATG9A. (**a**) HeLa cells were immunostained for endogenous ATG9A and VPS35 and subjected to confocal microscopy. Magnified areas are shown on the right of the pictures. (**b**) HeLa cells were transfected with ATG9A-GFP for 24 h, and subsequently fixed, immunostained for endogenous WASH1 and subjected to confocal microscopy. (**c**) HeLa cells were transfected with ATG9A-GFP as in **b**, but immunostained instead for endogenous FAM21. (**d**) HeLa cells stably expressing GFP-VPS35 WT and D620N were depleted of endogenous VPS35 using 40 nM of siRNA, and subsequently immunostained for TGN46 and endogenous ATG9A and subjected to confocal microscopy. (**e**) Colocalization between TGN and ATG9A is expressed in terms of the Pearson’s Coefficient. *n*=25 cells (WT) and 33 cells (D620N). Error bars represent s.e.m. and **P=0.01 by 2-tailed Student’s *t*-test. Scale bars in (**a**–**d**), 10 μm.

We observed that some ATG9A colocalized with VPS35, which is known to reside primarily on sorting endosomes, suggesting that ATG9A may traffic through compartments at which retromer acts (Fig. 6a). A proximity ligation assay, which yields a signal when two proteins of interest are within 40 nm of each other, indicated that ATG9 and VPS35 are found in the same compartment (Supplementary Fig. 5a). A signal is observed in untransfected cells, as well as cells stably expressing WT and D620N VPS35. The signal, although weak when compared with the signal obtained for anti-VPS35 and anti-VPS26, is specific and does not occur when anti-VPS35 is used alone. Furthermore, ATG9A colocalizes in part with WASH complex components at endosomes, thus suggesting that its trafficking may be perturbed by the WASH complex mislocalization associated with the VPS35 D620N mutation (Fig. 6b,c and Supplementary Fig. 5b).

In cells stably expressing WT GFP-VPS35, ATG9A colocalized with both the TGN marker TGN46 and VPS35 (Fig. 6d, Supplementary Fig. 6a, Supplementary Movies 1,2). However, the D620N cells showed increased colocalization between ATG9A and the TGN (Fig. 6e). In the D620N cells, the morphology of the TGN—and with it, ATG9A—changed dramatically from clusters to elongated perinuclear structures that were often circular or semicircular in shape. The aberrant TGN and ATG9A morphology in D620N cells was maintained under starvation conditions, and was present regardless of whether or not endogenous VPS35 has been depleted. Throughout a 2 h starvation period, ATG9A gradually dispersed from its basal perinuclear location in WT cells and fragmentation of the TGN was observed, consistent with previous reports^40^ (Supplementary Fig. 6b). However, in D620N-expressing cells, ATG9A and the TGN were maintained in the perinuclear area at all time points and did not undergo fragmentation. Aberrant Golgi morphology was confirmed with an independent TGN marker, Golgin-97, as well as the cis-Golgi marker GM130 (Supplementary Fig. 7a,b). The gross morphological changes of the TGN and ATG9A and their increased colocalization both suggest that normal trafficking of ATG9A is perturbed in D620N-expressing cells, and perhaps ATG9A is trapped in an abnormal perinuclear compartment.

**Figure 7:**
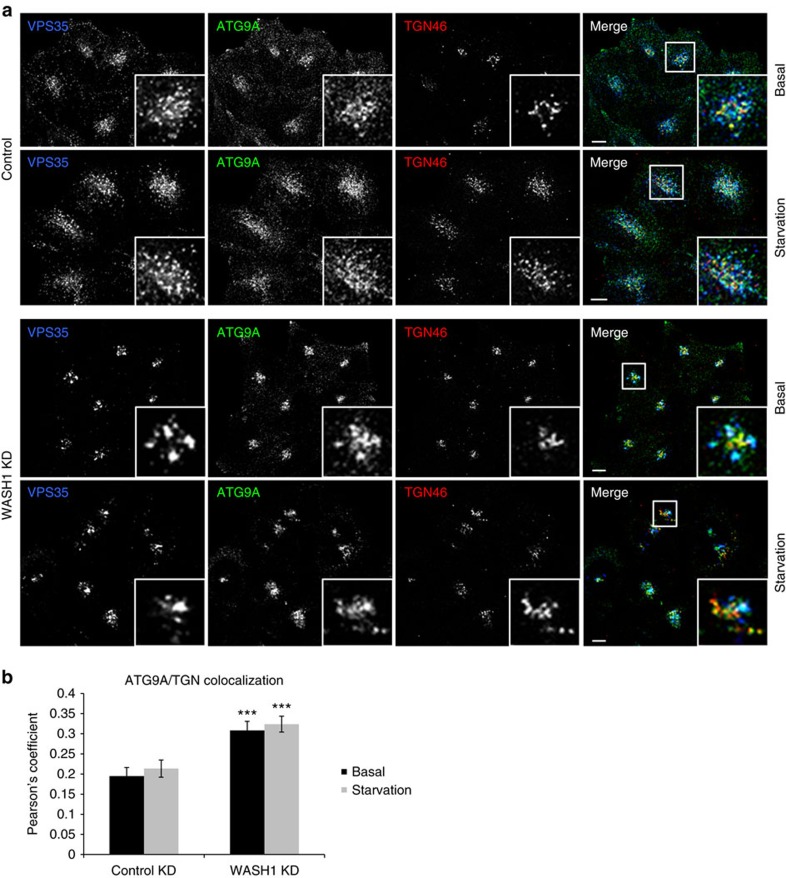
WASH1 depletion affects trafficking and localization of ATG9A. (**a**) HeLa cells depleted of WASH1 with two successive siRNA treatments were either starved in Hank’s balanced salt solution for 1 h or kept in full medium. Following fixation, cells were immunostained for TGN46, ATG9A and VPS35 and subjected to confocal microscopy. Representative pictures with magnified areas are shown. Scale bar, 10 μm. (**b**) Colocalization between TGN and ATG9A is expressed in terms of the Pearson’s coefficient. Number of cells analysed: 38 (control basal), 46 (knockdown basal), 36 (control starved), 53 (knockdown starved). Error bars represent s.e.m. and ***P=0.0005 (basal) and *P*=0.0004 (starved) by 2-tailed Student’s *t*-test.

Upon knockdown of WASH1 itself, colocalization of ATG9A with TGN46 once again was increased in the knockdown cells when compared with control (Fig. 7a,b, Supplementary Fig. 8a and Supplementary Movies 3–6). TGN46 and ATG9A morphology were also altered, although instead of elongated structures, we observed that ATG9A and TGN46 resided in tight clusters and remained so even upon starvation. This contrasts with control cells, where ATG9A noticeably dispersed upon starvation, and some fragmentation of the TGN46 marker was observed as well. Once again, other markers confirm an alteration of Golgi and TGN morphology (Supplementary Fig. 8b,c). While the phenotype of WASH1-depleted cells does not perfectly mimic the morphological disturbance observed with VPS35 D620N, it is important to note that the PD mutation does not abolish all WASH complex binding to retromer, and it also does not result in a depletion of WASH1 levels. Thus, the scenarios are not identical, but both serve to illustrate that ultimately, the activity of correctly localized WASH1 is necessary for proper ATG9A localization and trafficking. These effects on ATG9A localization may contribute to the defective autophagosome formation caused by the D620N mutation, as previous studies have demonstrated that genetic and pharmacological manipulations that impede ATG9A redistribution from perinuclear compartments inhibit autophagy^39^.

**Figure 8:**
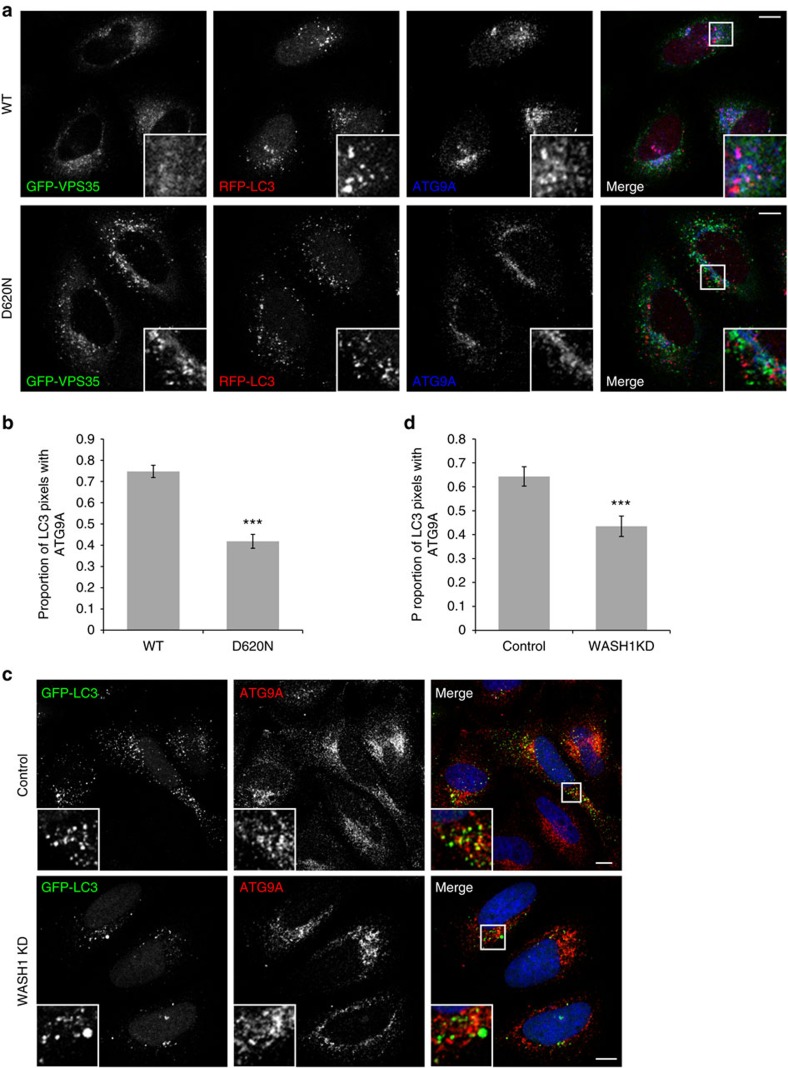
VPS35 D620N and WASH1 depletion impair ATG9A trafficking to autophagosomes. (**a**) HeLa cells stably expressing GFP-VPS35 WT and D620N were transfected with mRFP-LC3 for 24 h, immunostained for endogenous ATG9A, and imaged by confocal microscopy. (**b**) Colocalization is expressed in terms of Mander’s coefficient M1 to indicate the proportion of LC3 intensities that also contain ATG9A intensities. A representative experiment of three independent experiments is shown, in which at least 33 cells were analysed per condition. Error bars indicate s.e.m., and ***P<0.001 by 2-tailed Student’s *t*-test. (**c**) HeLa cells depleted of WASH1 were transfected with GFP-LC3 for 24 h, immunostained for endogenous ATG9A, and imaged by confocal microscopy. (**d**) Colocalization expressed as M1, as in **b**. A representative experiment of two independent experiments is shown, in which at least 24 cells were analysed per condition. Error bars indicate s.e.m., and ***P<0.001 by 2-tailed Student’s *t*-test. Scale bars in (**a**,**c**), 10 μm. In **b**,**d**, colocalization was measured for the entire area of the cell slice in each image. In order to ensure that results were not confounded by diffuse nuclear staining often observed in the LC3 channel, the analyses were repeated with the nuclei excluded, and all of the results remained statistically significant.

ATG9A acts early in the autophagy pathway and traffics to compartments that become LC3-positive to interact with phagophores and autophagosomes^41^. ATG9A is thought to deliver membrane to growing phagophores, and its depletion has detrimental effects on autophagy initiation^41^,^42^. In cells expressing WT GFP-VPS35, many of the LC3-positive autophagic structures also contained ATG9A (Fig. 8a,b, Supplementary Fig. 9a and Supplementary Movies 7,8). However, a significantly smaller proportion of LC3 vesicles contained ATG9A in cells expressing the D620N-mutant form of VPS35. WASH1 depletion had a similar effect, as knockdown of this protein resulted in fewer GFP-LC3 puncta containing ATG9A (Fig. 8c,d, Supplementary Fig. 9b and Supplementary Movies 9,10). Our results indicate that the VPS35 D620N mutation and WASH1 depletion both impair ATG9A trafficking to autophagosomes.

Although ATG9A mislocalization is a likely contributor to the autophagosome formation impairment seen in VPS35 D620N cells and WASH1-depleted cells, it is unlikely to be the only mechanism by which autophagy is compromised. We examined another early key autophagy protein, ATG16L1, and found no gross alterations in the distribution or appearance of mStrawberry-ATG16L1-positive vesicles in either VPS35 D620N cells or WASH1 knockdown conditions (Supplementary Fig. 10) However, it is interesting to note that in VPS35 D620N cells, we observed a decrease in the proportion of ATG16L1 vesicles containing ATG9A, echoing the phenotype observed with the diminished ability of LC3 vesicles to acquire ATG9A. A similar, although non-significant trend was observed upon WASH1 knockdown. Recent work has suggested that ATG9A and ATG16L1 vesicles, which enter the cell via distinct endocytic pathways, coalesce in recycling endosomes^43^. In fact, the recycling endosome marker RAB11 also shows altered morphology in both VPS35 D620N cells and WASH1-depleted cells (Supplementary Fig. 11).

**Figure 9:**
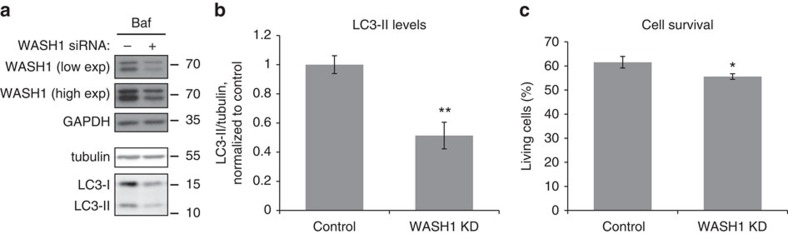
WASH1 depletion decreases neuronal cell survival. (**a**) SH-SY5Y cells were depleted of WASH1, treated with bafilomycin A1 and examined for LC3-II, tubulin, WASH1 and GAPDH levels. Blots shown are representative of two independent experiments in triplicate. (**b**) Quantification of the representative experiment in triplicate shown in (**a**), of two independent experiments. Error bars indicate s.e.m. **P=0.0058 by 1-tailed Student’s *t*-test. (**c**) WASH1-depleted SH-SY5Y cells were trypsinized, stained with propidium iodide and analysed by flow cytometry. Living cells, not stained with propidium iodide, are shown as a percentage of total cells. The graph depicts a representative experiment in triplicate out of two independent experiments, in which at least 10,000 cells were analysed in each replicate. Error bars indicate s.e.m. *P=0.045 by 1-tailed Student’s *t*-test.

Autophagy is known to protect against pro-apoptotic insults, and autophagy disruption predisposes to cell death^44^. A previous study indicated that the D620N VPS35 mutation is associated with impaired protection of neuronal cells from the mitochondrial toxin MPP+ (ref. 45). We found that knockdown of WASH1 in SH-SY5Y neuroblastoma cells decreases autophagosome formation and increases cell death (Fig. 9).

## Discussion

The retromer complex recruits the WASH complex to endosomes through VPS35 binding to the FAM21 protein^4^,^11^,^12^,^13^. Our data suggest that a major molecular effect of the VPS35 D620N mutation is to reduce retromer association with the WASH complex as well as FKBP15, without affecting CSC assembly. The WASH complex functions to promote F-actin patch formation on endosomes and its activity is associated with endosomal protein sorting of multiple cargo proteins^10^. Indeed, we find that cells with the D620N VPS35 mutation have decreased membrane-associated WASH complex components and phenotypes such as impaired cell spreading and GLUT-1 mislocalization, seen in cells with compromised WASH-complex function^9^,^11^.

The most obvious disease-relevant phenotype that we associated with the D620N mutation is impaired autophagosome formation. Similar autophagy defects were seen in WASH1 knockdown cells or in cells overexpressing the FAM21 tail, which inhibits association of the WASH complex with VPS35 by competition^11^,^12^,^13^. While disturbed retromer–WASH complex association may have multiple consequences for autophagosome formation, we have shown that the D620N mutation, as well as WASH1 depletion, affects ATG9A localization and trafficking. We recently showed that ATG9A is internalized from the plasma membrane by clathrin-mediated endocytosis and travels via early endosomes to the recycling endosome^43^. The data shown here indicate that the VPS35 D620N mutation or WASH1 depletion result in ATG9A becoming trapped in a perinuclear compartment positive for a TGN marker. It is, however, difficult to distinguish between proteins in the TGN and recycling endosomes even by electron microscopy^46^, raising the possibility that the ATG9A is retained in the recycling endosome and is unable to continue its normal trafficking to phagophores and autophagosomes. In fact, the recycling endosome marker RAB11 also shows perturbed morphology in both VPS35 D620N-expressing and WASH1-depleted cells. Alterations in ATG9A trafficking result in a smaller proportion of LC3-positive autophagic structures acquiring ATG9A and likely contributes to the decreased autophagy phenotype. Other perturbations that cause similar ATG9A trafficking defects also impair autophagosome formation^40^,^47^,^48^,^49^. Interestingly, autophagosome formation deficits associated with defective ATG9A trafficking are also seen with α-synuclein overexpression, which models other autosomal-dominant forms of PD caused by α-synuclein gene duplications^27^. Although no obvious changes were observed in the distribution of another important early autophagy protein, ATG16L1, it is possible that other autophagy components might be affected.

Recent studies have reported that the D620N mutation in VPS35 can alter CIMPR localization and impair cathepsin D trafficking, resulting in lysosomal enlargement^50^,^51^. We found no alterations in CIMPR or LAMP1 staining between cells stably expressing WT or D620N-mutant VPS35. Furthermore, the decreases in LC3-II levels in the VPS35 D620N cells point to defective autophagosome formation, rather than degradation, which would be expected with lysosome perturbations. It is nonetheless possible that the VPS35 D620N mutation leads to additional phenotypes—including those affecting lysosomal function and/or phenotypes specific to neuronal cells—that are not explored here.

Our data suggest that the D620N mutation is a partial loss of function mutation of just one role of VPS35: that of binding the WASH complex and FKBP15, and not a generalized VPS35 hypomorph. Unlike a knockdown of VPS35 that abolishes retromer function and leads to mislocalization of many proteins that traffic between the endosome and either Golgi or cell surface, the D620N mutation appears to affect only those aspects of retromer function that are linked to the WASH complex. The GLUT-1 protein is especially dependent on the activity of the WASH complex for its localization^7^,^35^ and hence localization of GLUT-1 is perturbed in cells expressing the D620N mutation. Similarly, cell spreading is also tightly linked to WASH complex function and the D620N cells exhibit a cell spreading defect^9^,^11^. The retromer complex can still function in endosome-to-Golgi retrieval without the WASH complex, as demonstrated in yeast where the WASH complex is not conserved and yet retromer works to retrieve Vps10p from endosomes to the Golgi.

Patients carrying this D620N mutation only manifest disease in their 6th decade^18^,^19^. If the D620N mutant were to drastically affect the localization of a great many membrane proteins, one would expect a more severe disease and earlier onset. Nonetheless, we cannot formally exclude that the D620N mutation has other effects in addition to impaired WASH complex binding and a resultant autophagy deficiency, and that some of these unexplored phenotypes could be due to a gain of function of the mutant protein.

The VPS35 mutation also exhibits defective binding to FKBP15 and our data are consistent with the hypothesis that the retromer CSC, WASH complex and FKBP15 form a tripartite complex. The precise function of FKBP15 remains to be determined and is likely to be multifaceted^52^,^53^. Unlike the WASH complex, FKBP15 is not conserved in either *Caenorhabditis elegans* or *Drosophila melanogaster*^4^. Our data suggest that FKBP15 is unlikely to be an important contributor to the autophagy phenotype. Nevertheless, it is conceivable that the loss of retromer–FKBP15 interaction resulting from the D620N mutation may contribute to the pathology of PD in other ways.

Interestingly, another PD gene, DNAJC13 (ref. 54), encodes a protein that has recently been shown to associate with the FAM21 protein of the WASH complex—the same subunit known to interact with VPS35 (ref. 55). Thus, the WASH complex associates with two known PD proteins, suggesting that WASH complex function may be especially important for the pathology of PD.

In conclusion, our data show that the D620N PD-causing VPS35 mutation impairs binding to the WASH complex and FKBP15. We found that loss of WASH complex binding was likely responsible for the impaired autophagosome formation resulting from the D620N VPS35 mutation. This autophagy defect was associated with abnormal ATG9A trafficking. Impaired autophagy is a highly plausible contributor to PD pathogenesis, as it would exacerbate many of the pathologies associated with PD, including protein aggregation, mitochondrial abnormalities, increased levels of reactive oxygen species and enhanced susceptibility to cell death.

## Methods

### Mammalian cell culture

HeLa and SH-SY5Y cells were maintained in DMEM containing 4,500 mg l^−1^ of glucose, and supplemented with 10% fetal bovine serum (FBS), 100 units ml^−1^ penicillin, 100 μg ml^−1^ streptomycin and 2 mM L-glutamine (all from Sigma-Aldrich) at 37 °C and 5% CO_2_. HeLa cells stably expressing GFP-VPS35 constructs, GFP-FAM21 tail or VPS29-GFP were generated by the Seaman Laboratory and cultured in DMEM containing 4,500 mg l^−1^ of glucose, and supplemented with 10% FBS, 100 units ml^−1^ penicillin, 100 μg ml^−1^ streptomycin, 2 mM L-glutamine and 500 μg ml^−1^ G418 (Gibco). Cell lines used in autophagy experiments were regularly tested for mycoplasma contamination and treated when appropriate. Starvation experiments were carried out in Hank’s balanced salt solution (Gibco). Transfections were performed in OptiMEM (Gibco) using Lipofectamine 2000 (Life Technologies) for siRNA and TransIT 2020 (Mirus Bio) for DNA, according to the manufacturer’s protocol. In large scale transfections of cells for IP experiments, polyethylenimine (Polysciences Inc.) was employed as the carrier following a protocol similar to Lipofectamine-mediated transfections as described previously^12^.

### RNA interference-mediated silencing

In IP experiments, the silencing of VPS35, WASH complex components and FKBP15 was achieved using siRNA following previously described knockdown protocols^11^. Predesigned siRNA were ordered from Thermo Fisher Scientific (siRNA IDs: nontargeting control D-01810-10; WASH1 ON-TARGETplus SMARTpool L-029524 (currently sold as L-190043-00); FAM21 ON-TARGETplus SMARTpool L-029678-01; FKBP15 ON-TARGETplus SMARTpool L-029587-01; KIAA1033 ON-TARGETplus SMARTpool L-026919-01; VPS35 single siRNAs J-010894-05, J-010894-07 (used in GFP-VPS35 stable cell lines), and J-010894-08). The siRNA were resuspended in 1 × siRNA buffer (Thermo Fisher, B-002000-UB-100). In Figs 1, 2, 3 and Supplementary Figs 1–3, control indicates that no siRNA was used, whereas in Figs 4, 5, 6, 7, 8, 9 and Supplementary Figs 4–11, control knockdown indicates that a nontargeting pool was used.

### DNA constructs

GFP-tagged VPS35 has been described previously^56^. The D620N mutation was engineered into murine VPS35 in pEGFP C1 using the QuikChange kit (Stratagene) and presence of the mutation was confirmed by DNA sequencing. The construct was subcloned into pmCherry to generate the mCherry-tagged version. The GFP-tagged FAM21 tail construct has also been described previously^11^. The first exon of the huntingtin protein with 74 polyglutamines, tagged with EGFP or HA at the N terminus, in pEGFP-C1 vector (GFP-Q74) or pHM6 vector (HA-Q74) has been described previously^23^,^57^. GFP-α-synuclein has been described previously^58^, as has mCherry-RAB11 (ref. 43). ATG9A-GFP was a kind gift of Yoshinori Takahashi and was described previously^40^. mStrawberry-ATG16L1, GFP-LC3 and mRFP-LC3 were kindly provided by Tamotsu Yoshimori and were described previously^59^,^60^. The mRFP-RAB5 construct was described by Vonderheit and Helenius^61^.

### Antibodies and reagents

Antibodies used in this study include rabbit anti-ATG9A (1:200 for IF, Abcam ab108338); mouse anti-EEA1 (1:200 for IF, Abcam ab70521); rabbit anti-FAM21 (1:200 for WB, generated in house, described previously^11^); rabbit anti-FAM21 (1:100–1:200 for IF, Santa Cruz, sc-137995); rabbit anti-FKBP15 (1:400–1:500 for WB, generated in house, described previously^4^); rabbit anti-FKBP15 (1:200 for IF, Abcam, ab14432); mouse anti-GAPDH (1:10,000 for WB, Abcam ab8245); mouse anti-GFP (1:10,000 for WB, Clontech, 632375); mouse anti-GM130 (1:100 for IF, BD Biosciences, 610822); mouse anti-Golgin-97 (1:200 for IF, Molecular Probes, A-21270); mouse anti-HA (1:500 for IF, Covance MMS-101P); rabbit anti-LC3 (1:2,000 for WB, Novus NB100-2220); rabbit anti-strumpellin (1:400 For WB, Santa Cruz, sc-87442); sheep anti-TGN46 (1:100 for IF, AbD Serotec AHP500); mouse anti-α-tubulin (1:15,000 for WB, Sigma T9026); rabbit anti-VPS26 (1:1,000 for WB, Abcam ab23892); two different rabbit anti-VPS26, described previously^3^, (one for IF, the other at 1:1,000 for WB); goat anti-VPS29 (1:5,000 for WB or 1:1,000 when used with 125I-protein A for detection Abcam ab10160), mouse anti-VPS35 (1:1,000 for WB, 1:200 for IF, Abcam ab57632), mouse anti-VPS35 (1:500 For WB, Santa Cruz, sc-374372), rabbit anti-VPS35 (1:400 for WB, as described previously^3^), rabbit anti-WASH1 (N terminus) (1:1,000 for WB, Millipore ABS73), rabbit anti-WASH1 (C terminus) (1:200 for IF, Millipore ABS72), and rabbit anti-WASH1 (1:300 for WB, Sigma, SAB4200372). Bafilomycin A1 was obtained from Sigma-Aldrich and used for 4 h at 400 nM. An equivalent volume of DMSO was used as a vehicle control.

### Western blotting

Detection for western blotting of native IP samples and accompanying lysates was accomplished using ^125^I-protein A and exposure to X-ray film as described previously^62^. In autophagy assays, after lysing cells in sample buffer (62.5 mM Tris pH 6.8, 2% w/v SDS, 10% glycerol, 50 mM DTT, 0.01% w/v bromophenol blue) western blot analysis was performed using standard techniques with an ECL western blotting substrate (Thermo Fisher Scientific) or with direct infrared fluorescence detection on an Odyssey Infrared Imaging System. For ECL western blotting, the following horseradish peroxidase-conjugated secondary antibodies were used: sheep anti-mouse (GE Healthcare, NA931V); donkey anti-rabbit (GE Healthcare, NA934V); rabbit anti-goat (Life Technologies, 61-1620). For infrared fluorescence detection, IRDye-conjugated secondary antibodies were obtained from LI-COR (926-32220, 926-32211, 926-32210). Whole blot images are presented in Supplementary Fig. 12.

### Native IP

Stably or transiently transfected HeLa cells in 140 mm tissue culture dishes were lysed on ice in 1 ml of lysis buffer (20 mM HEPES-KOH, pH 7.2, 50 mM K-acetate, 200 mM sorbitol, 2 mM EDTA, 0.1% Triton X-100 with protease inhibitors, or PBS with 1% Triton X-100 and protease inhibitors, as indicated). Lysates were centrifuged, precleared with 50 μl of a 25% slurry of protein A-sepharose for 30 min and subsequently incubated with antibodies for 90 min. The mix was incubated with protein A-sepharose for 60 min, after which sepharose beads were recovered by centrifugation, washed four times in lysis buffer, dessicated in a SpeedVac (Eppendorf), resuspended in SDS–PAGE sample buffer and boiled for 5 min.

### Membrane association assay

Determination of the amount of membrane-associated strumpellin and FKBP15 was performed using a simple fractionation assay described previously^4^,^11^,^63^.

### Immunofluorescence

Unless otherwise indicated, cells were cultured for immunofluorescence microscopy on glass coverslips, fixed with 4% paraformaldehyde in PBS for 5 min, permeabilized with 0.1% Triton X-100 in PBS for 5 min, and washed with 0.2% glycine in PBS twice for 5 min each. Coverslips were blocked in 10% FBS in PBS, and subsequently incubated with primary antibodies for 2 h, washed three times with PBS and incubated with Alexa Fluor-conjugated secondary antibodies (Life Technologies) for 30 min. Samples were mounted using ProLong Gold Antifade reagent with 4',6-diamidino-2-phenylindole (Life Technologies) and imaged at 37 °C with a Zeiss LSM 710 laser confocal microscope using a Plan-Apochromat × 63/1.40 Oil DIC M27 lens and ZEN acquisition software, or in the case of Supplementary Fig 7b, with a Zeiss LSM510 laser confocal microscope using a × 100 oil immersion lens and LSM510 acquisition software (Carl Zeiss). Images shown represent individual confocal slices, unless stated otherwise. Compressed z-stacks and three-dimensional movies were created with Volocity software (Perkin Elmer). Colocalization was determined by calculating the Pearson’s coefficient or Mander’s coefficient M1/M2 by tracing individual cells with Volocity software (Perkin Elmer). Thresholds were kept constant within each experiment. For Fig. 3 and Supplementary Fig. 2, the following protocol was employed: cells were fixed for 15 min using 4% paraformaldehyde in PBS, permeabilized with 0.1% Triton X-100 in PBS for 10 min prior to blocking with 3% bovine serum albumin (BSA) in PBS for 20 min. Cells were labelled with antibodies diluted in blocking buffer (3% BSA in PBS) for 1 h, washed and then incubated with Alexa Fluor-conjugated secondary antibodies (Life Technologies) diluted in blocking buffer for a further hour. The cells were mounted using ProLong Gold Antifade reagent (Life Technologies) and viewed at room temperature using a Plan-Apochromat × 63 lens with oil immersion on a Zeiss AxioPlan epifluorescence microscope equipped with a Hamamatsu CCD camera and SimplePCI acquisition software (Hamamatsu). To quantify the amount of endosomally localized FAM21 and FKBP15, cells were imaged using a Cellomics ArrayScan VTI HCS Reader and the Spot Detector application (Thermo Fisher Scientific) and FKBP15 and FAM21 spot intensity was normalized to GFP-VPS35 and VPS26 signals. The Cellomics Spot Detector application was also used to quantify the number of GFP-LC3 and mRFP-LC3 vesicles per cell.

### Proximity ligation assay

The proximity ligation assay kit was obtained from Sigma and used according to manufacturer's instructions. Briefly, cells were seeded onto 13 mm coverslips and allowed to grow in culture for 24 h. After fixation with 4% paraformaldehyde in PBS, permeabilization with 0.1% Triton X-100 in PBS and blocking with 3% BSA in PBS, the cells were incubated with anti-VPS35 and either anti-VPS26 or anti-ATG9A. Following incubation with the primary antibodies, the cells were incubated with secondary antibodies conjugated to oligonucleotide primers. The primers were ligated and then rolling circle amplification was used to create a reaction product that is observable by microscopy due to hybridization of fluorescently labelled nucleotides. Successful production of a DNA product requires that the primary antibodies bind their respective antigens and reside within 40 nm of each other. Coverslips were mounted on slides and imaged by epifluorescence microscopy, as described in the previous section.

### Cell spreading

Cells were plated on glass coverslips and fixed in 4% paraformaldehyde in PBS for 5 min at 2, 4 and 6 h after plating. Cells were permeabilized in 0.1% Triton X-100 in PBS for 5 min, and incubated with Alexa Fluor 594-conjugated phalloidin for 30 min to mark the cell periphery. The cell perimeter was measured using Cellomics ArrayScan VTI HCS Reader and the Cell Spreading application (Thermo Fisher Scientific). Bright-field images of cells were taken immediately prior to fixation with an EVOS FL Cell Imaging System (Life Technologies).

### Aggregation

Cells were plated on glass coverslips and transfected with GFP-Q74 or HA-Q74 for 24 h before fixation with 4% paraformaldehyde in PBS. Coverslips expressing GFP-Q74 were mounted ProLong Gold Antifade reagent with 4',6-diamidino-2-phenylindole (Life Technologies), while coverslips with HA-Q74 were subjected to immunofluorescence against HA before mounting. Using a Nikon Eclipse E600 fluorescence microscope, an experimenter blinded to the identity of the slides scored the percentage of transfected cells containing aggregates. Experiments were performed in triplicate and a minimum of 200 cells were counted in each replicate.

### α-synuclein degradation

Cells expressing GFP-VPS35 wild type or D620N were plated in six-well plates and transfected with 1 μg GFP-α-synuclein A53T and 0.5 μg GFP DNA per well. Forty-eight hours after transfection, cells were lysed in sample buffer and the lysates analysed by western blotting. GFP-α-synuclein levels were expressed as a ratio with GFP levels.

### Cell survival

SH-SY5Y neuroblastoma cells were depleted of WASH1 using two rounds of siRNA treatment, as described above. Seventy-two hours after the second treatment, trypsinized cells and their growth medium from the last 24 h (containing any detached cells) were spun down and the cells resuspended in 500 μl of PBS on ice. Viability was determined by staining with propidium iodide ReadyProbes reagent (Life Technologies). Cells were analysed by a BD FACSCalibur flow cytometer (BD Biosciences) and Flow-Jo software (Treestar) in the FL2 channel, and cells positive for propidium iodide were classified as dead.

### Statistics

Significance levels for comparisons between groups were determined by Student’s *t*-tests.

## Author contributions

E.Z. and M.N.J.S. planned, performed and analyzed the majority of the experiments reported herein with additional experimental contributions from K.M., M.J.-S., S.Y.B. and M.E.H. D.C.R. planned and analyzed experiments. E.Z., M.N.J.S. and D.C.R. wrote the manuscript.

## Additional information

**How to cite this article**: Zavodszky, E. *et al.* Mutation in VPS35 associated with Parkinson’s disease impairs WASH complex association and inhibits autophagy. *Nat. Commun.* 5:3828 doi: 10.1038/ncomms4828 (2014).

## Supplementary Material

Supplementary Figures Supplementary Figures 1-12

Supplementary Movie 1 HeLa cells stably expressing GFP-VPS35 wild-type were depleted of endogenous VPS35 using 40 nM of siRNA, and subsequently immunostained for TGN46 and endogenous ATG9A and subjected to confocal microscopy. 3D z-stack visualization is shown.

Supplementary Movie 2 HeLa cells stably expressing GFP-VPS35 D620N were depleted of endogenous VPS35 using 40 nM of siRNA, and subsequently immunostained for TGN46 and endogenous ATG9A and subjected to confocal microscopy. 3D z-stack visualization is shown.

Supplementary Movie 3 HeLa cells were treated with control siRNA and kept in full medium. Following fixation, cells were immunostained for TGN46, ATG9A, and VPS35 and subjected to confocal microscopy. 3D z-stack visualization is shown.

Supplementary Movie 4 HeLa cells were treated with control siRNA and starved for one hour in HBSS. Following fixation, cells were immunostained for TGN46, ATG9A, and VPS35 and subjected to confocal microscopy. 3D z-stack visualization is shown.

Supplementary Movie 5 HeLa cells depleted of WASH1 with two successive siRNA treatments were kept in full medium. Following fixation, cells were immunostained for TGN46, ATG9A, and VPS35 and subjected to confocal microscopy. 3D z-stack visualization is shown.

Supplementary Movie 6 HeLa cells depleted of WASH1 with two successive siRNA treatments and starved in HBSS for one hour. Following fixation, cells were immunostained for TGN46, ATG9A, and VPS35 and subjected to confocal microscopy. 3D z-stack visualization is shown.

Supplementary Movie 7 HeLa cells stably expressing GFP-VPS35 wild-type were transfected with mRFP-LC3 for 24 hours, immunostained for endogenous ATG9A, and imaged by confocal microscopy. 3D z-stack visualization is shown.

Supplementary Movie 8 HeLa cells stably expressing GFP-VPS35 D620N were transfected with mRFP-LC3 for 24 hours, immunostained for endogenous ATG9A, and imaged by confocal microscopy. 3D z-stack visualization is shown.

Supplementary Movie 9 HeLa cells were treated with control siRNA and subsequently transfected with GFP-LC3 for 24 hours, immunostained for endogenous ATG9A, and imaged by confocal microscopy. 3D z-stack visualization is shown.

Supplementary Movie 10 HeLa cells depleted of WASH1 with two successive siRNA treatments were then transfected with GFP-LC3 for 24 hours, immunostained for endogenous ATG9A, and imaged by confocal microscopy. 3D z-stack visualization is shown.
