# Supplementary material for: Mutation in VPS35 associated with Parkinson’s disease impairs WASH complex association and inhibits autophagy
Source: Nat Commun. 2014 May 13;5:3828. doi: 10.1038/ncomms4828 (PMC4024763; doi:10.1038/ncomms4828)
Supplement: Supplementary Figures — 1-12 [file ncomms4828-s1.pdf]

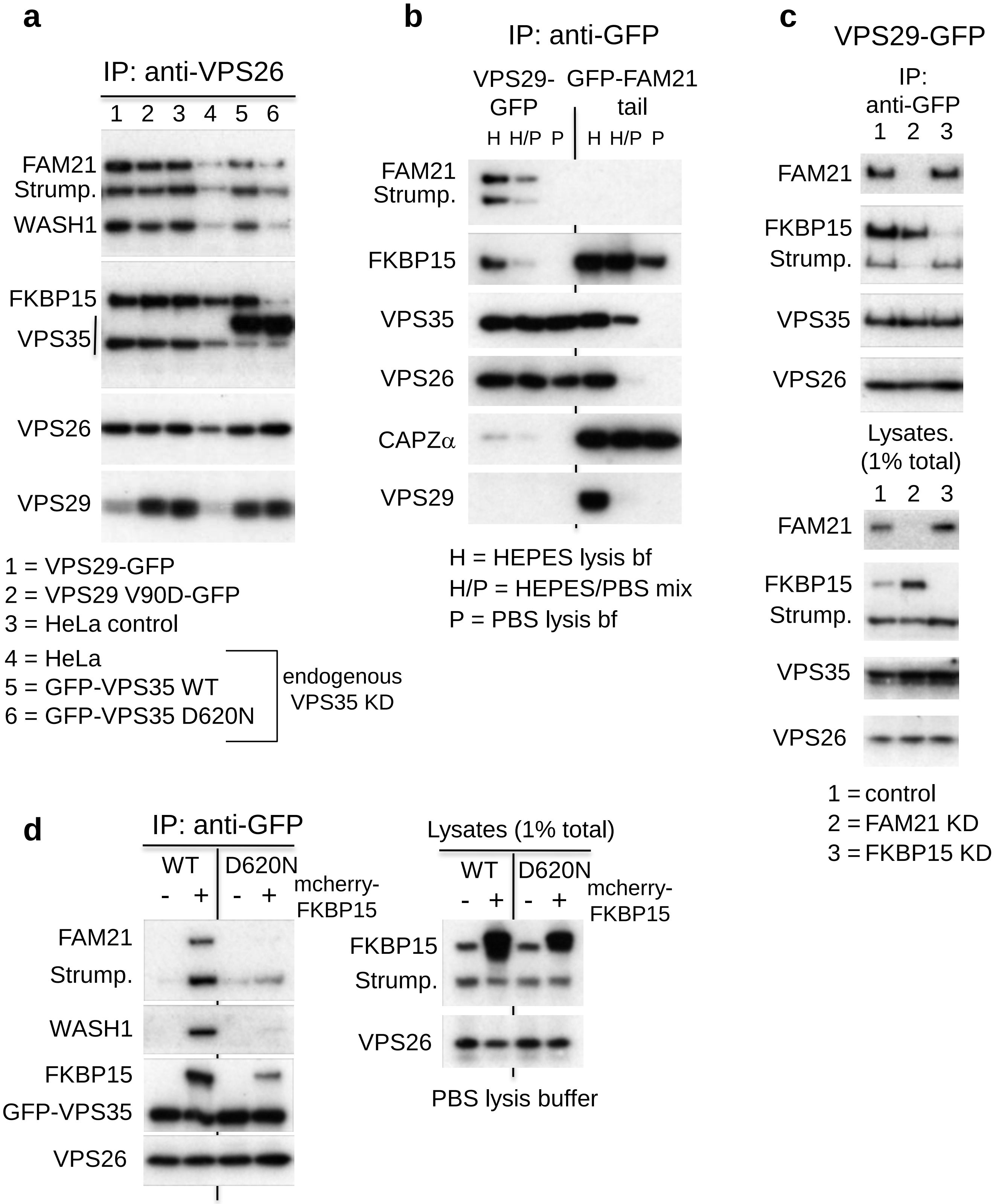

Supplementary Figure 1. Interactions of the retromer CSC. (**a**) Cells stably expressing GFP-tagged wild-type or point mutant versions of VPS29 or VPS35 along with untransfected HeLa cells were lysed and immunoprecipitated with anti-VPS26 antisera. (**b**) Cells stably expressing VPS29-GFP or GFP-FAM21 tail were lysed using either the low stringency HEPES buffer (H), the higher stringency PBS buffer (P) or the mixed HEPES/PBS buffer (H/P) as in Figure 2. Lysates were treated with anti-GFP to recover the respective GFP-tagged protein. (**c**) FKBP15 or FAM21 expression was abolished with siRNA treatment in the VPS29-GFP expressing cells. Cells were lysed and the VPS29-GFP recovered by native IP. (**d**) Cells stably expressing either wild-type GFP-VPS35 or the D620N mutant were transiently transfected with mCherry-FKBP15 or a control plasmid 48 hours prior to lysis in the higher stringency PBS buffer. GFP-VPS35 and associated proteins were recovered by native IP.

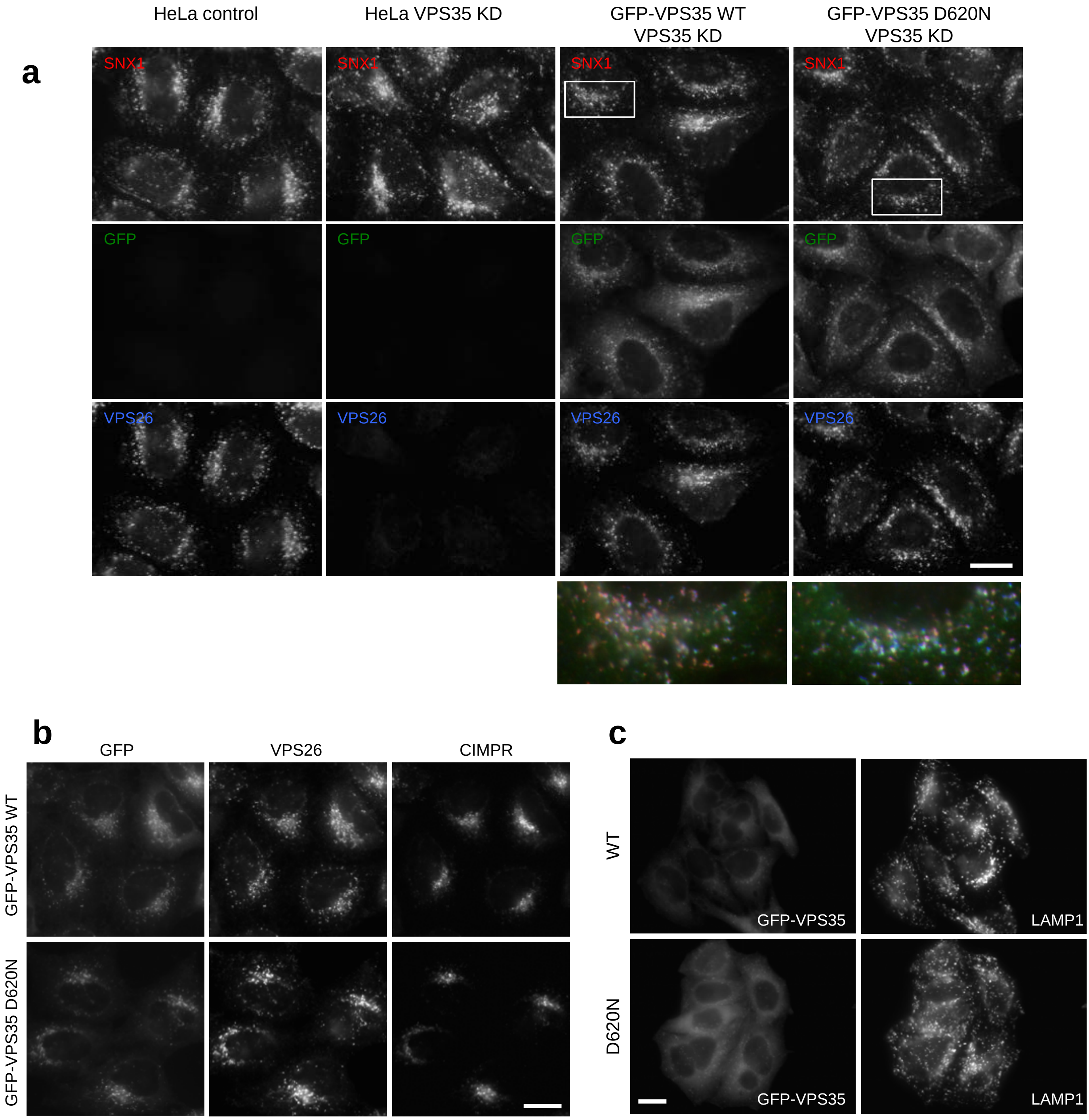

Supplementary Figure 2. The VPS35 D620N mutant localizes normally to endosomes. (**a**) Untransfected HeLa cells and HeLa cells stably expressing GFP-VPS35 wild-type (WT) and D620N were treated with siRNA to silence endogenous VPS35 expression. 72 hours post-transfection, cells were fixed and stained with antibodies against GFP, SNX1 and VPS26. The loss of VPS35 results in loss of VPS26. Expression of either WT or D620N VPS35 rescues the VPS26 staining and both WT and D620N VPS35 appear to localize to SNX1-positive structures. (**b**) Cells expressing either wild-type GFP-VPS35 or D620N were treated with siRNA to silence expression of endogenous VPS35. 72 hours post-transfection, cells were fixed and labeled with antibodies against VPS26 and CIMPR. (**c**) HeLa cells stably expressing GFP-VPS35 wild-type (WT) and D620N were fixed and labeled with antibody against LAMP1. LAMP1 staining did not appear markedly different from cells expressing WT VPS35. Scale bars in (a)-(c) = 20 µm.

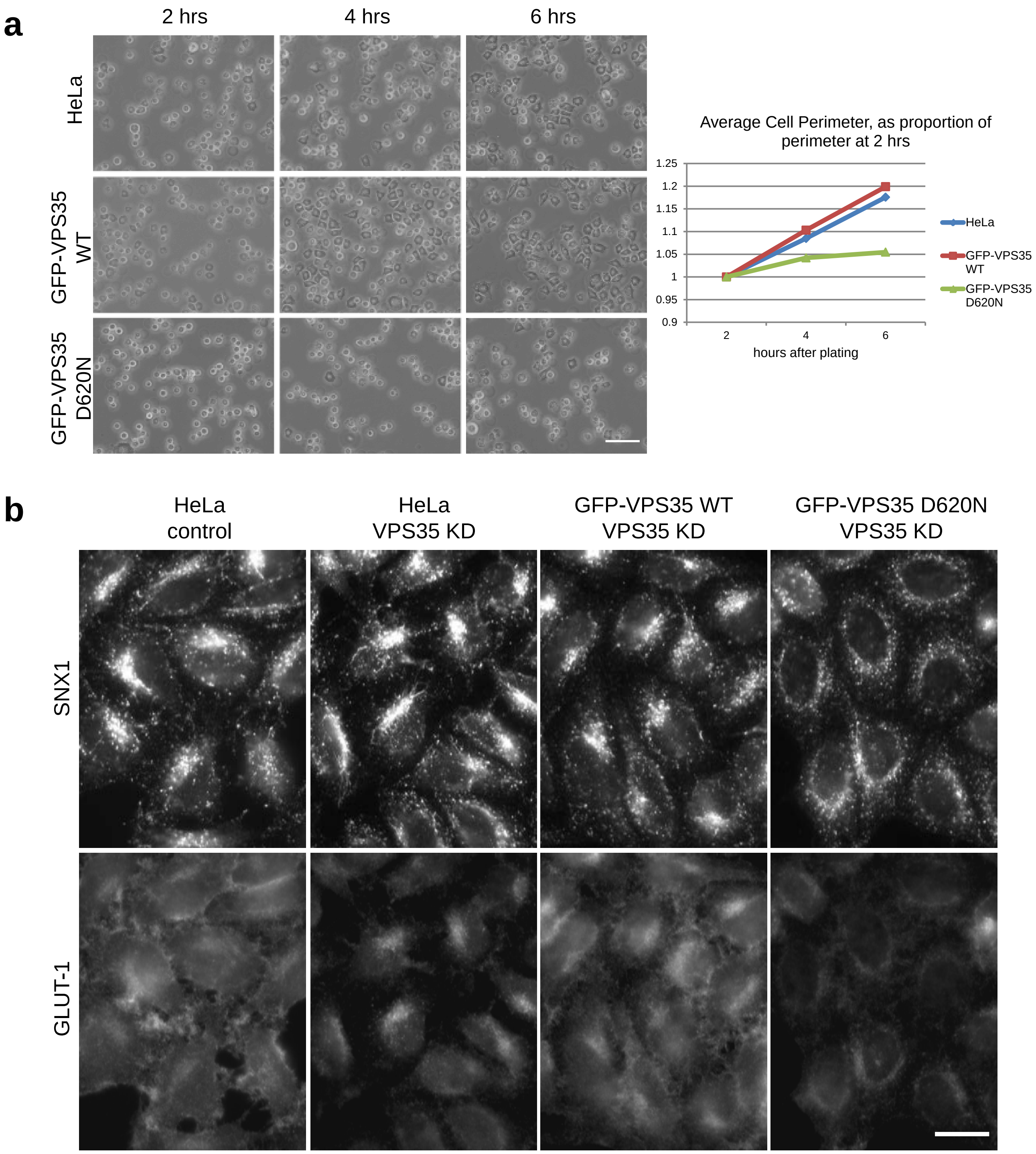

Supplementary Figure 3. The VPS35 D620N mutant impacts on WASH-associated phenotypes. (**a**) Untransfected HeLa cells and HeLa cells stably expressing GFP-VPS35 wild-type (WT) and D620N were plated on glass coverslips and fixed at 2, 4, and 6 hours after plating. Representative images are shown for each condition. Scale bar = 100 µm. The graph represents the average cell perimeter quantified by Cellomics automated fluorescence microscopy. A minimum of 5000 cells per coverslip were analysed. (**b**) Untransfected HeLa cells and HeLa cells stably expressing GFP-VPS35 wild-type (WT) and D620N were treated with siRNA to silence endogenous VPS35 expression. 72 hours post-transfection, cells were fixed and stained with antibodies against SNX1 and GLUT-1. Cells were selected for imaging by viewing in the SNX1 channel without viewing the GLUT-1 staining so as to avoid biasing the observed GLUT-1 localisation. The knockdown of VPS35 shifts the localization of GLUT1 to intracellular structures. The expression of GFP-tagged WT VPS35 can rescue GLUT-1 localisation but the D620N mutant displays GLUT-1 localisation more similar to a VPS35 KD in untransfected HeLa cells.

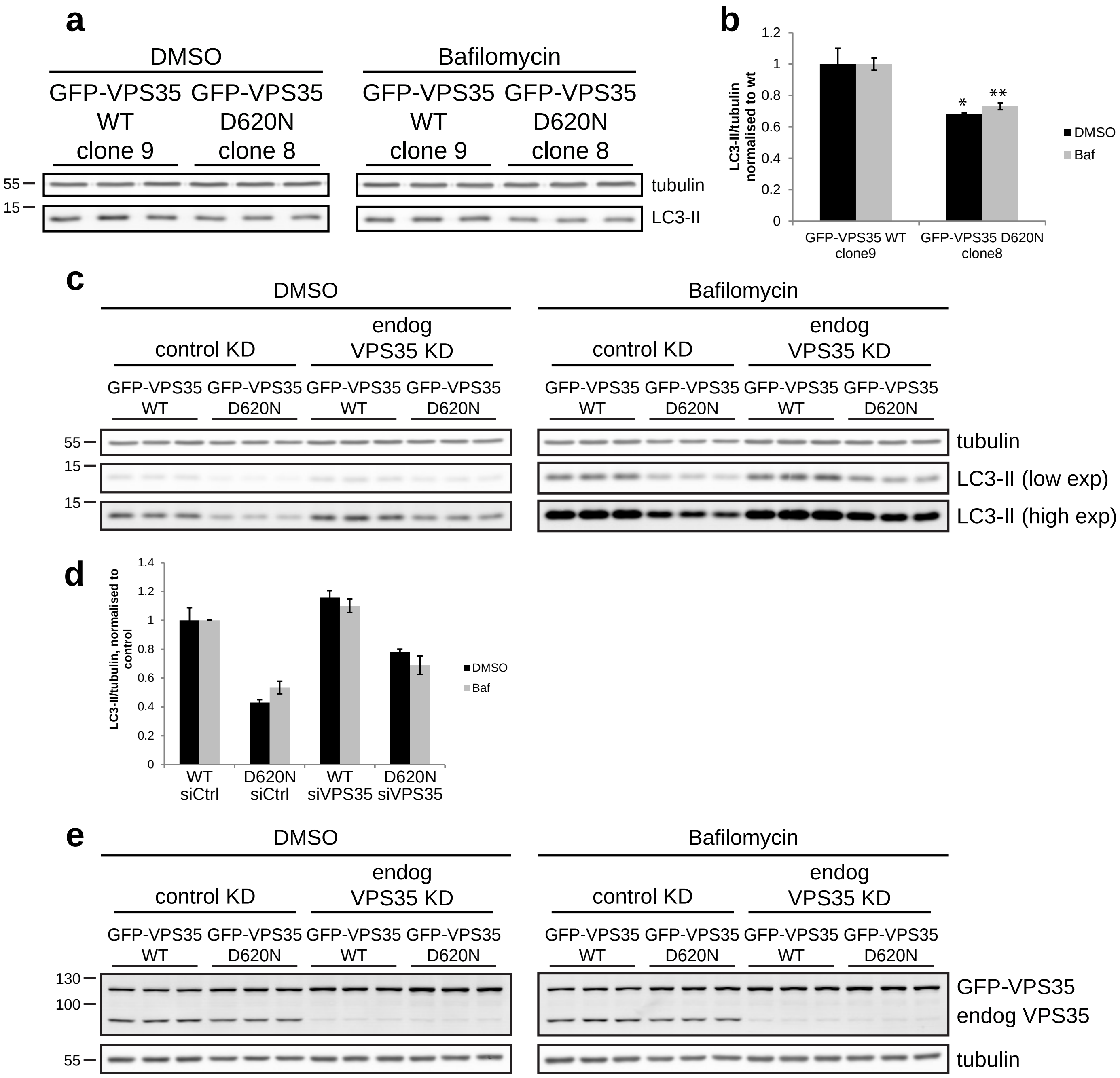

Supplementary Figure 4. VPS35 impairs autophagy in independent clones and is unaffected by knockdown of the endogenous protein. (**a**) HeLa cells stably expressing GFP-VPS35 wild-type (WT) and D620N were derived from clones different than those in Fig. 1. The cells were treated with Bafilomycin A1, lysed, and assessed for LC3-II and tubulin levels as described previously. A representative blot is shown. (**b**) The experiment in triplicate shown in (a) is quantified with LC3-II levels normalised to tubulin and expressed as a ratio of levels in wild-type. Error bars indicate SEM. * indicates p=0.03 and ** indicates p=0.004 by 2-tailed Student's t-test. (**c**) The original clones of HeLa cells stably expressing GFP-VPS35 WT and D620N were treated once with 40 nM siRNA to silence endogenous human VPS35 without affecting the GFP-tagged murine construct. Cells were treated with Bafilomycin A1 as previously described and assessed for LC3-II and tubulin levels. (low exp: low exposure: high exp: high exposure) (**d**) Quantification of the experiment in triplicate shown in (c). Error bars indicate SEM. The p-values (all two-tailed) between wild-type and D620N cells with a control knockdown are 0.003 and 0.0004 for DMSO and Baf, respectively. The p-values between wild-type and D620N cells depleted of endogenous VPS35 are 0.002 and 0.007 for DMSO and Baf, respectively. The p-value for the comparison of D620N cells with and without depletion of endogenous VPS35 in DMSO is 0.0002. All other comparisons between cells with and without depletion of endogenous VPS35 are nonsignificant.

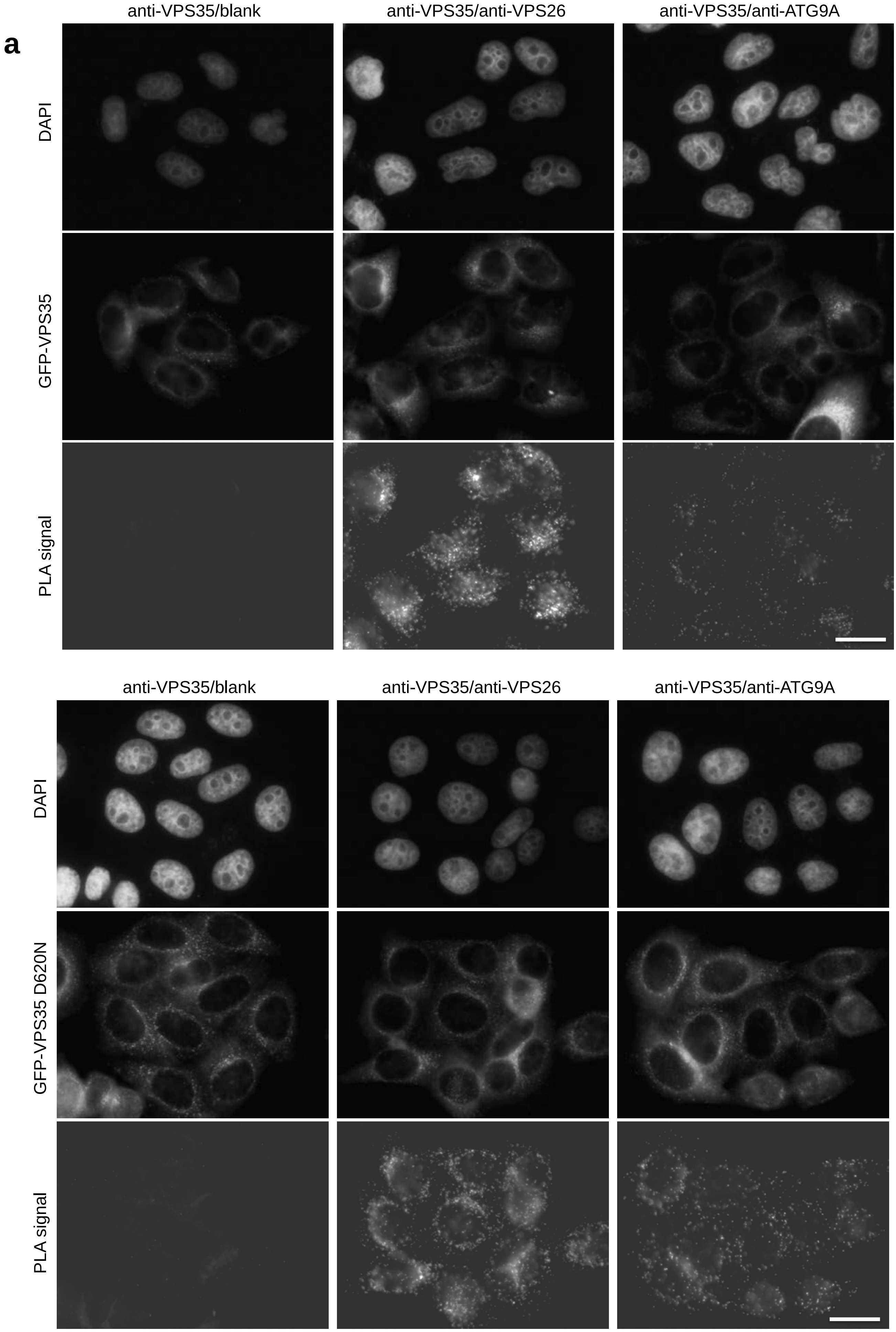

Supplementary Figure 5 (continued on next page)

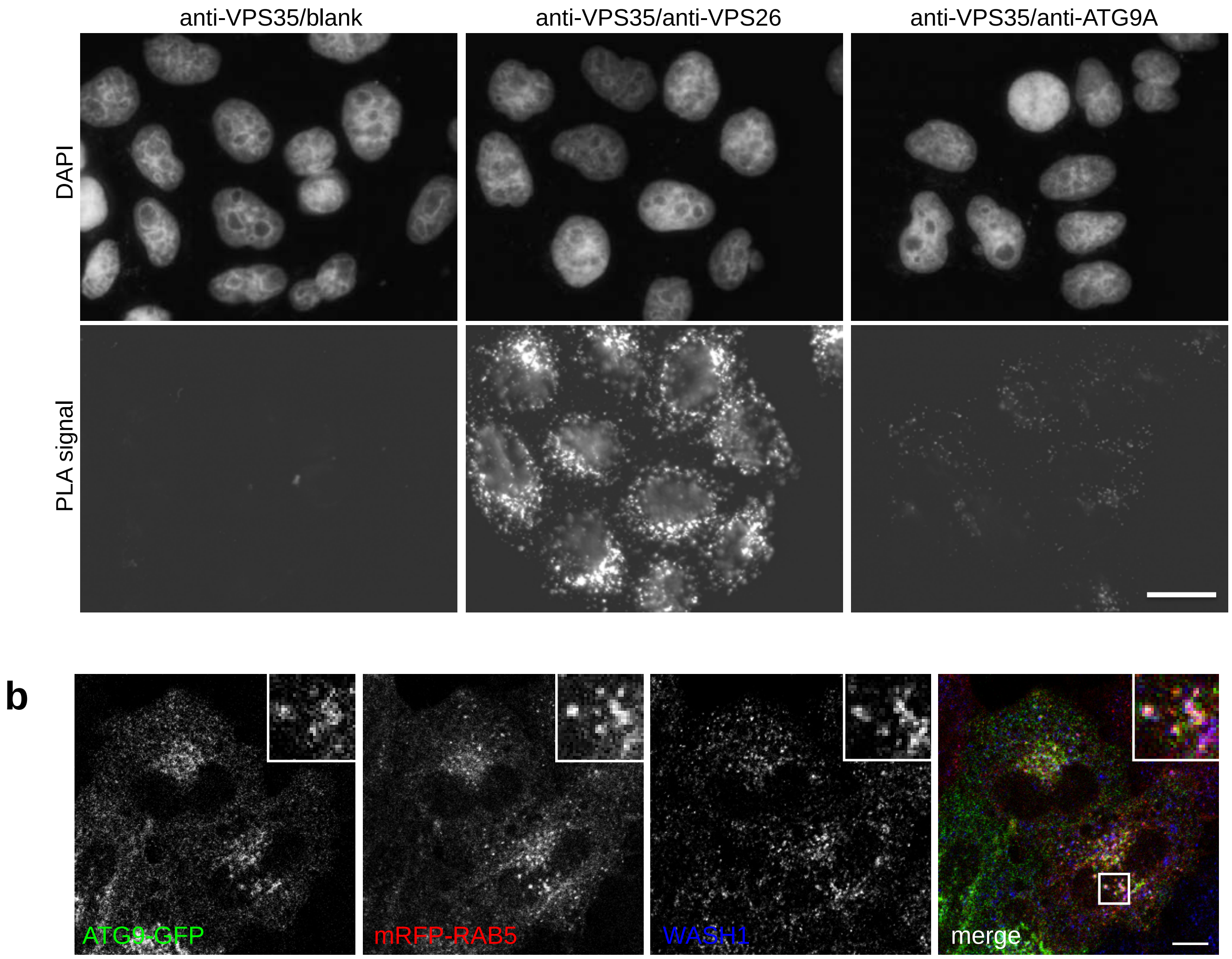

Supplementary Figure 5. ATG9A resides in the same compartment as VPS35 and the WASH complex. (**a**) Untransfected HeLa cells, or cells stably expressing GFP-VPS35 or GFP-VPS35 D620N were fixed and analysed using the proximity ligation assay (PLA), with primary antibodies as indicated. The cells were imaged by epifluorescence microscopy. Scale bar = 20 µm. (**b**) HeLa cells were transfected with ATG9A-GFP and mRFP-RAB5, immunostained for endogenous WASH1, and subjected confocal microscopy. Scale bar = 10 µm.

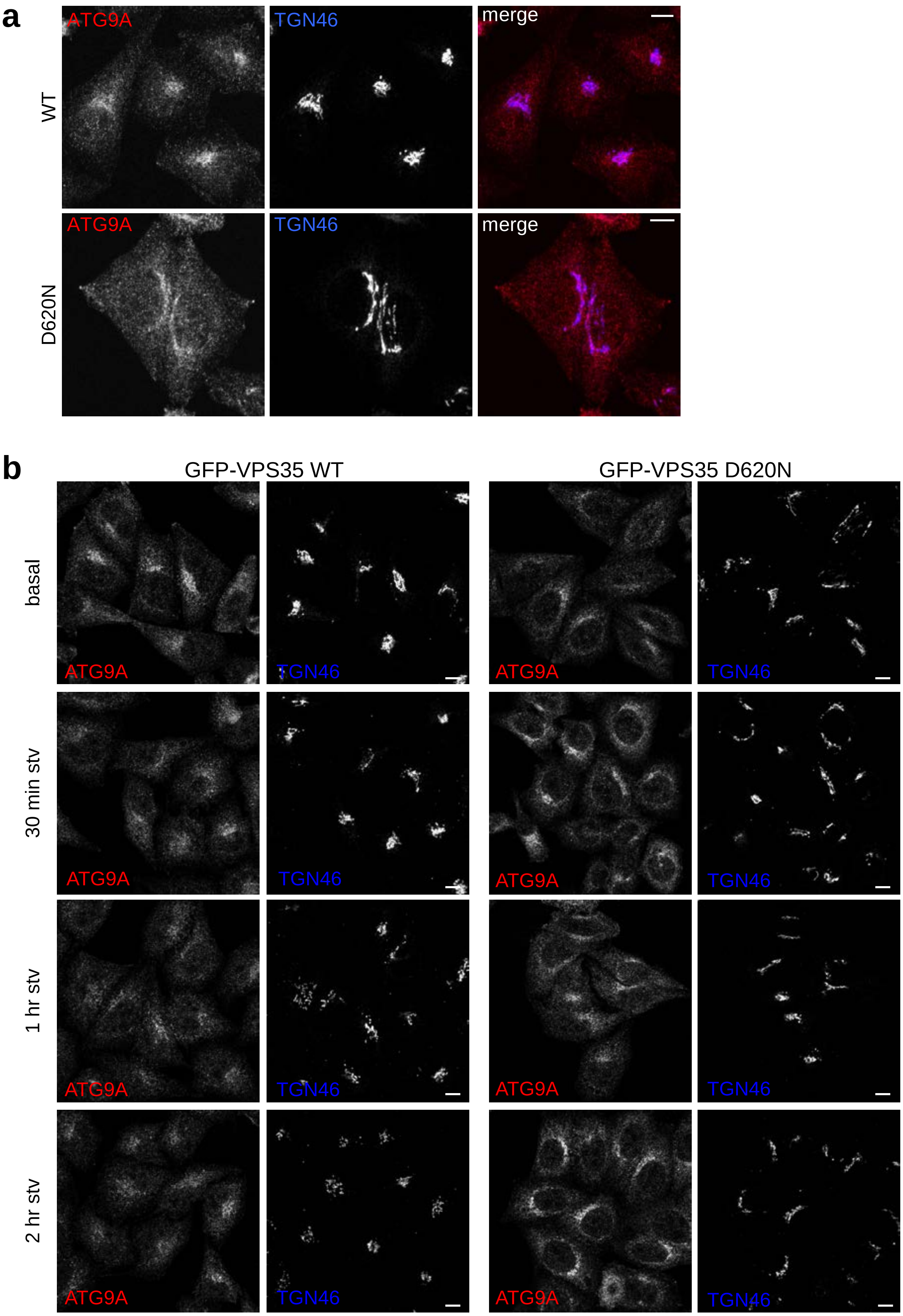

Supplementary Figure 6. VPS35 D620N affects trafficking and localization of ATG9A.
(**a**) HeLa cells stably expressing GFP-VPS35 wild-type (WT) and D620N were depleted of endogenous VPS35 using 40 nM of siRNA, and subsequently immunostained for TGN46 and endogenous ATG9A and subjected to confocal microscopy, as in Fig. 6d. Compressed z-stack images are shown. (**b**) HeLa cells stably expressing GFP-VPS35 wild-type (WT) and D620N were starved in HBSS for the time indicated or kept in full medium (“basal”). Following fixation, cells were immunostained for TGN46 and endogenous ATG9A and subjected to confocal microscopy. Confocal slices are shown.
Scale bars in (a) and (b) = 10 µm.

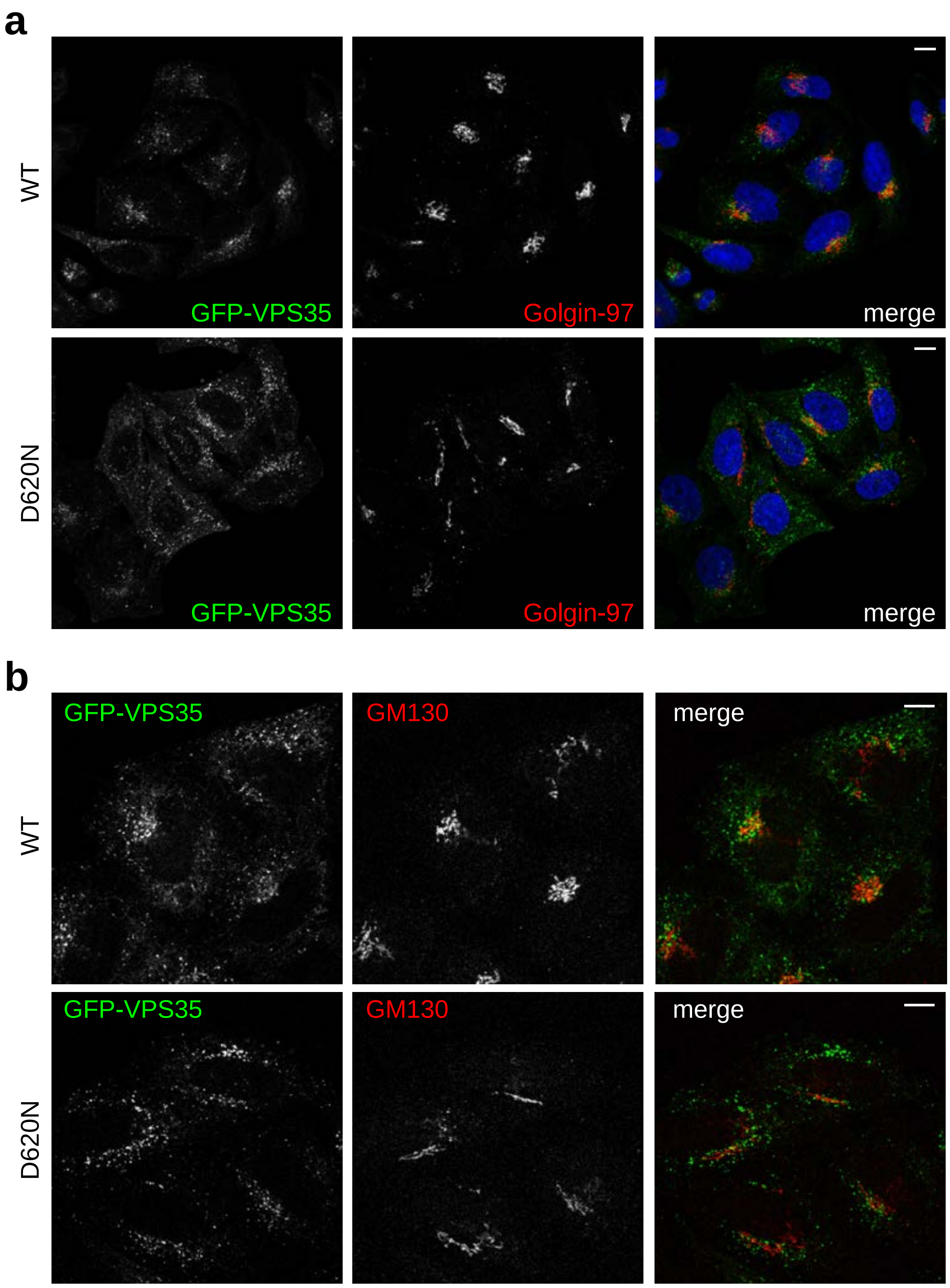

Supplementary Figure 7. VPS35 D620N alters Golgi and TGN morphology. (**a**) HeLa cells stably expressing GFP-VPS35 wild-type (WT) and D620N were depleted of endogenous VPS35 using 40 nM of siRNA, and subsequently immunostained for Golgin-97 and subjected to confocal microscopy. (**b**) HeLa cells stably expressing GFP-VPS35 wild-type (WT) and D620N were fixed and immunostained for GM130 and subjected to confocal microscopy. Scale bars in (a) and (b) = 10 μm.

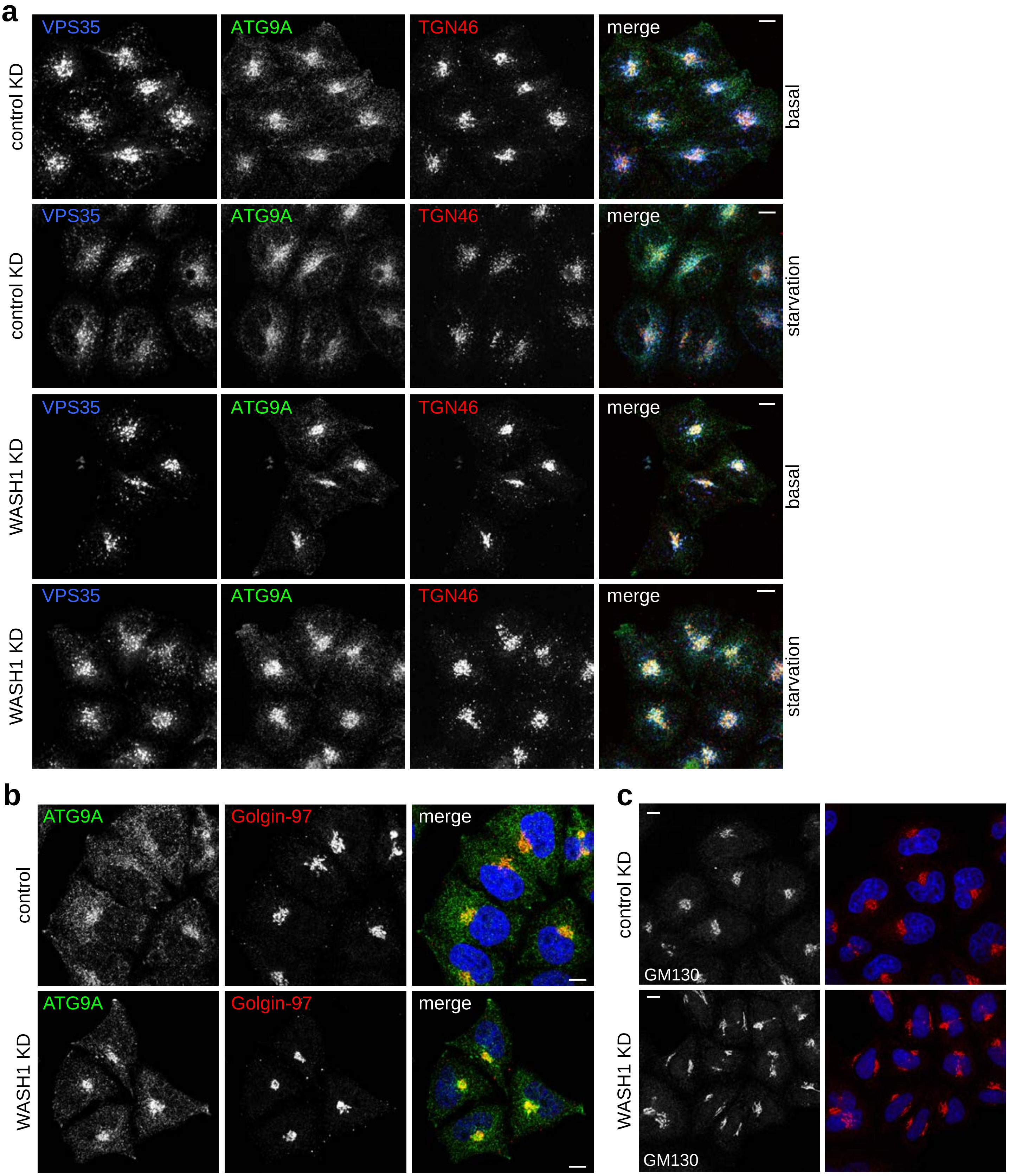

Supplementary Figure 8. WASH1 depletion affects ATG9 localization, as well as TGN and Golgi morphology. (**a**) HeLa cells depleted of WASH1 with two successive siRNA treatments were either starved in HBSS for one hour or kept in full medium. Following fixation, cells were immunostained for TGN46, ATG9A, and VPS35 and subjected to confocal microscopy, as in Fig. 7a. Compressed z-stack images are shown. (**b**) HeLa cells were depleted of WASH1 and fixed as in (a), and immunostained for ATG9A and Golgin-97. Confocal slices are shown. (**c**) HeLa cells were depleted of WASH1 and fixed as in (a), and immunostained for GM130. Confocal slices are shown. Scale bars in (a)-(c) = 10 µm.

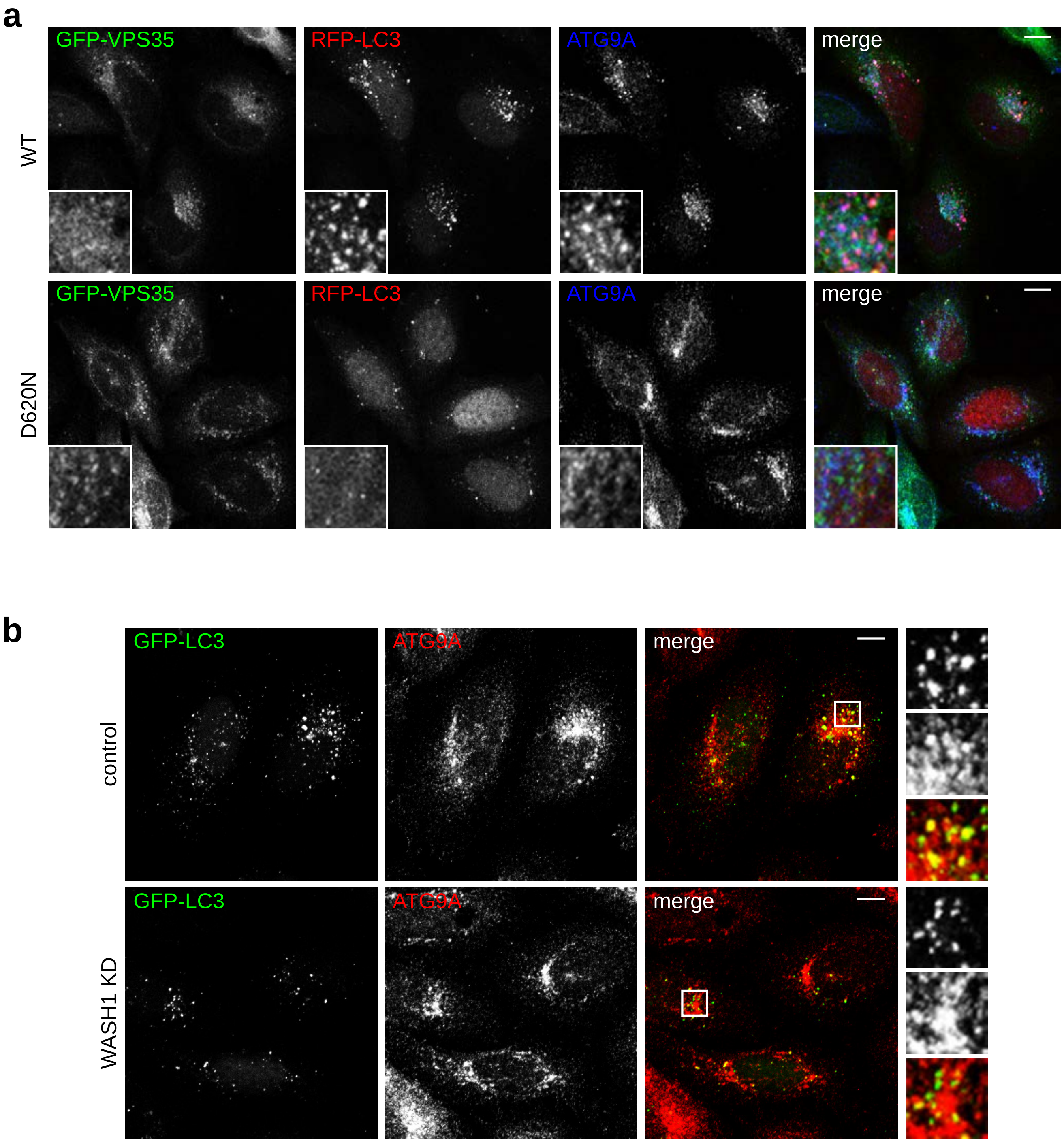

Supplementary Figure 9. VPS35 D620N and WASH1 depletion impair ATG9A trafficking to autophagosomes. (**a**) HeLa cells stably expressing GFP-VPS35 wild-type and D620N were transfected with mRFP-LC3 for 24 hours, immunostained for endogenous ATG9A, and imaged by confocal microscopy. Compressed z-stack images are shown. (**b**) HeLa cells depleted of WASH1 with two successive siRNA treatments were then transfected with GFP-LC3 for 24 hours, immunostained for endogenous ATG9A, and imaged by confocal microscopy. Compressed z-stack images are shown. Scale bars in (a) and (b) = 10 µm.

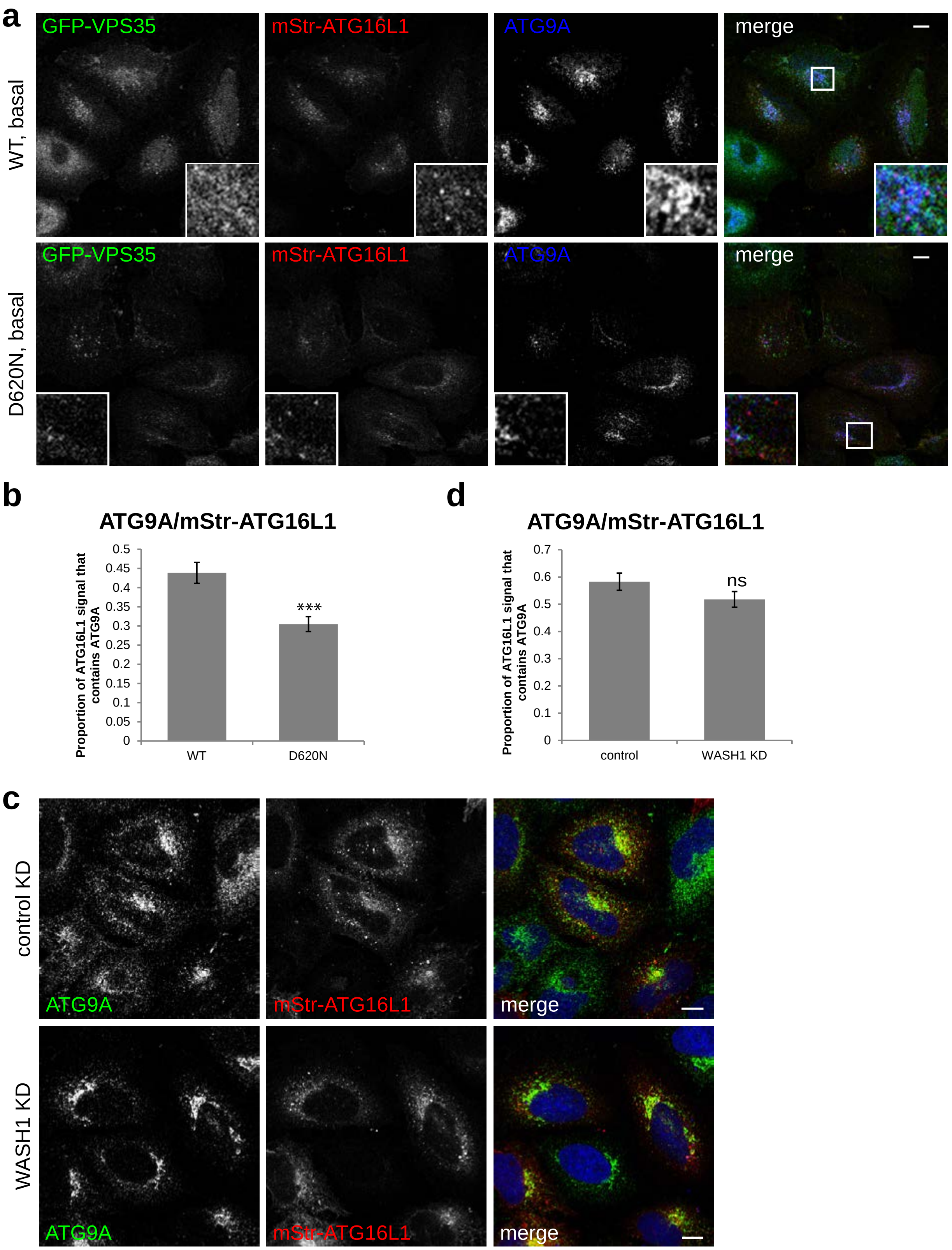

Supplementary Figure 10. VPS35 D620N and WASH1 depletion do not alter ATG16 distribution. (**a**) HeLa cells stably expressing GFP-VPS35 wild-type and D620N were transfected with mStrawberry-ATG16L1 for 24 hours, immunostained for endogenous ATG9A, and imaged by confocal microscopy. (**b**) Colocalization is expressed in terms of Mander's coefficient M1 to indicate the proportion of ATG16L1 intensities that also contain ATG9A intensities. n=34 cells (WT) and 27 cells (D620N). Error bars represent SEM and *** indicates p=0.00036 by 2-tailed Student's t-test. (**c**) HeLa cells depleted of WASH1 with two successive siRNA treatments were then transfected with mStrawberry-ATG16L1 for 24 hours, immunostained for endogenous ATG9A, and imaged by confocal microscopy. (**d**) Colocalization expressed as Mander's coefficient, as in (b). n=18 cells (control) and 19 cells (WASH1 knockdown). Error bars indicate SEM, p=0.14 by 2-tailed Student's t-test. Scale bars in (a) and (c) = 10 μm.

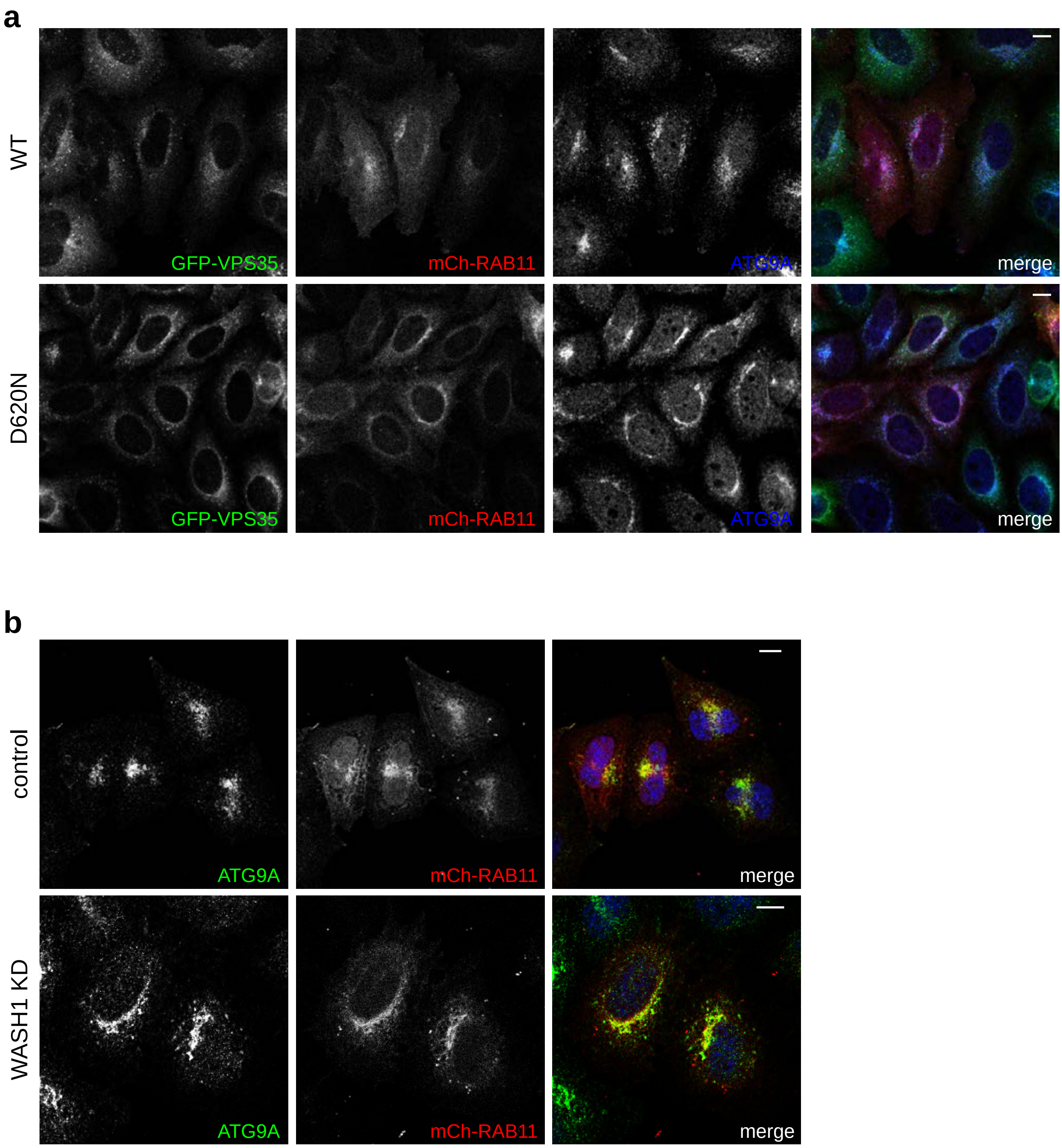

Supplementary Figure 11. VPS35 D620N and WASH1 depletion alter recycling endosome morphology. (**a**) HeLa cells stably expressing GFP-VPS35 wild-type and D620N were transfected with mCherry-RAB11 for 24 hours, immunostained for endogenous ATG9A, and imaged by confocal microscopy. (**b**) HeLa cells depleted of WASH1 with two successive siRNA treatments were then transfected with mCherry-RAB11 for 24 hours, immunostained for endogenous ATG9A, and imaged by confocal microscopy. Scale bars in (a) and (b) = 10 µm.

Figure 1b

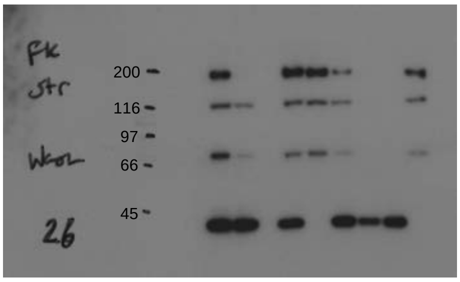

WB: FKBP15, Strumpellin, WASH1, VPS26

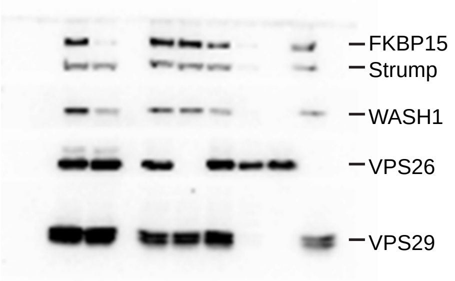

WB: FKBP15, Strumpellin, WASH1, VPS26, VPS29 (phosphoimager image)

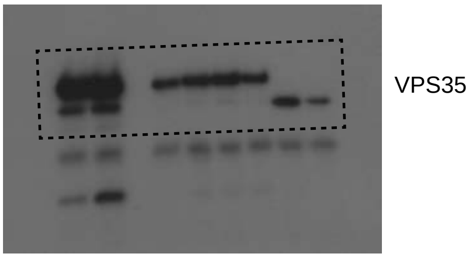

WB: VPS35

Figure 1c

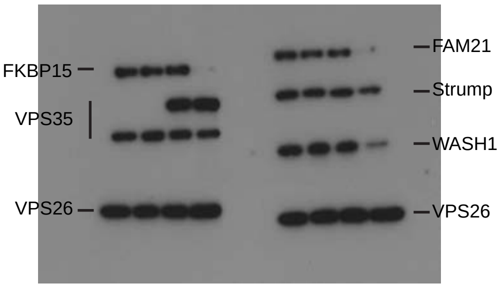

WB: FKBP15, VPS35, VPS26, FAM21, Strumpellin, WASH1 (higher exp.)

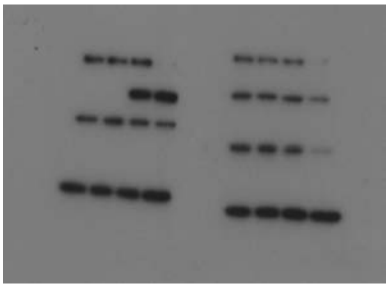

WB: FKBP15, VPS35, VPS26, FAM21, Strumpellin, WASH1 (lower exp.)

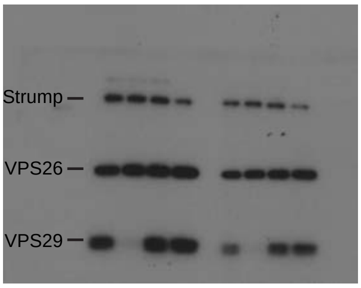

WB: Strumpellin, VPS26, VPS29

Figure 1d

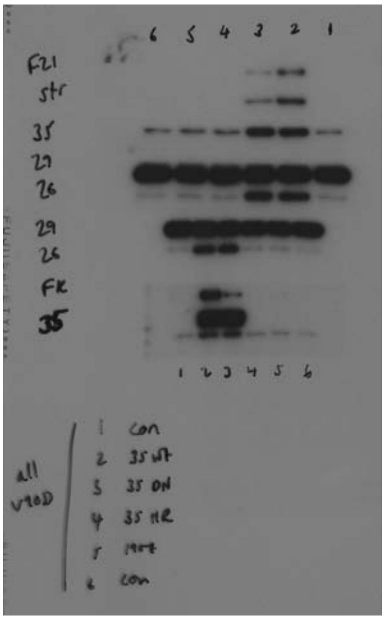

WB: FAM21, Strumpellin, FKBP15, VPS35, VPS26, VPS29

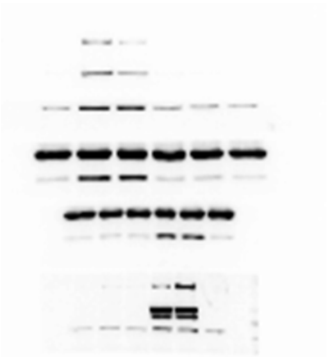

WB: FAM21, Strumpellin, FKBP15, VPS35, VPS26, VPS29 (phosphoimager image)

Figure 2a

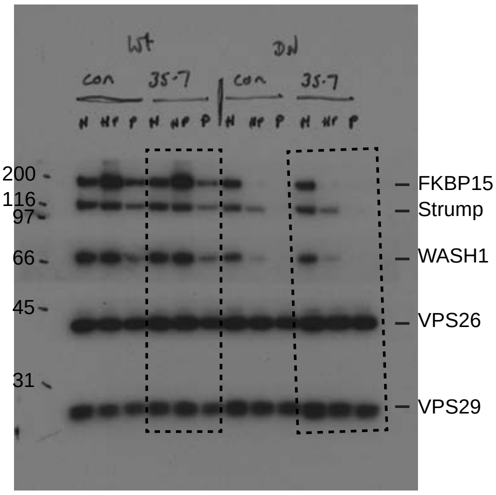

WB: FKBP15, Strumpellin, WASH1, VPS26, VPS29

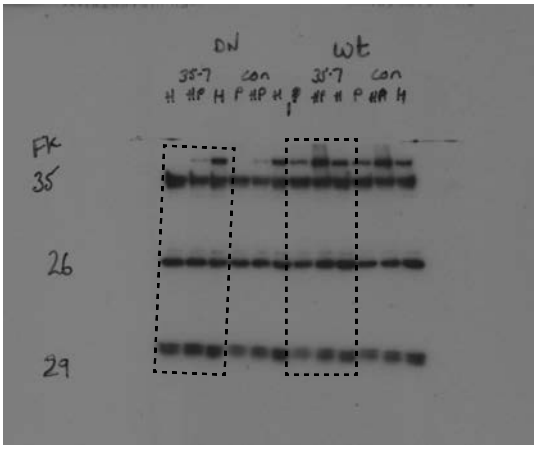

WB: FKBP15, VPS35, VPS26, VPS29

Figure 2a, continued

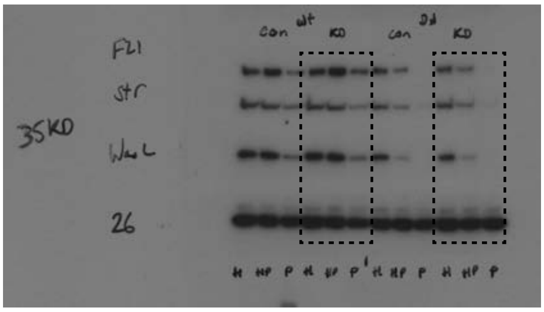

WB: FAM21, Strumpellin, WASH1, VPS26

Supplementary Figure 12. Full scans of uncropped blots

Figure 2b

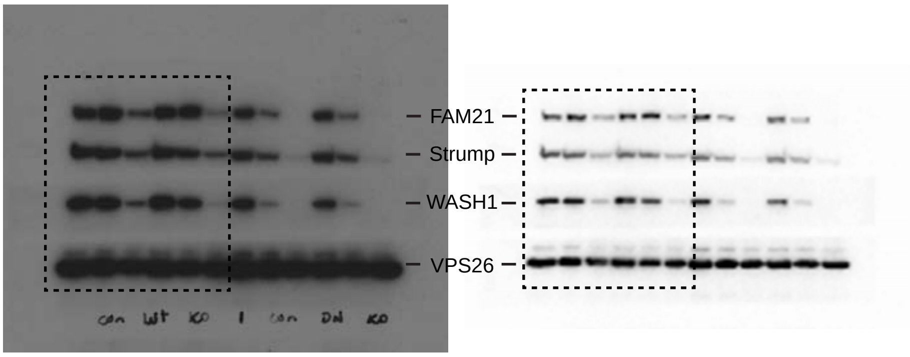

WB: FAM21, Strumpellin, WASH1, VPS26
(right image is from phosphoimager)

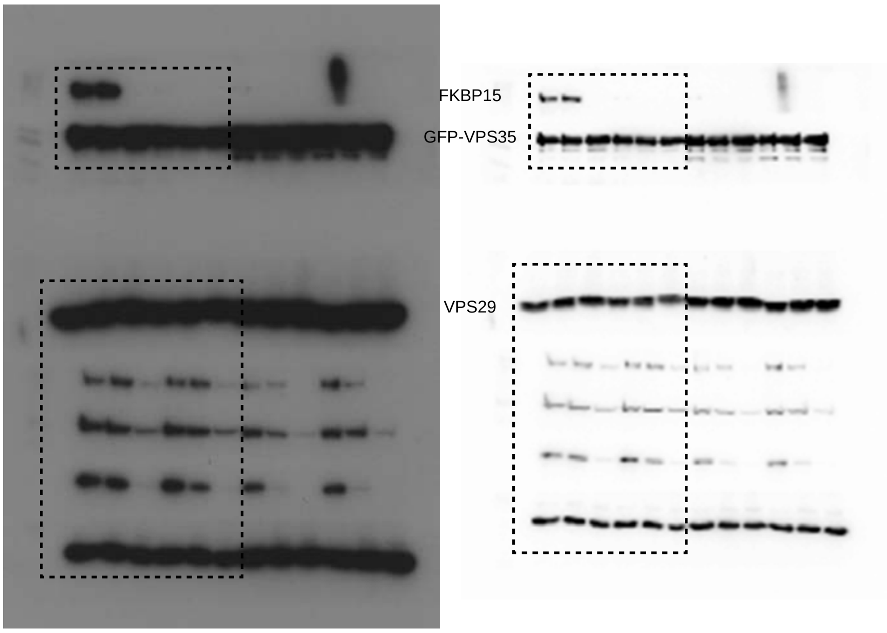

WB: FKBP15, VPS35, VPS29
(right image is from phosphoimager)

Figure 2c

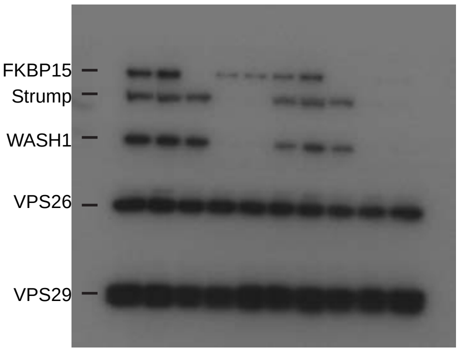

WB: FKBP15, Strumpellin, WASH1, VPS26, VPS29
(lower exp)

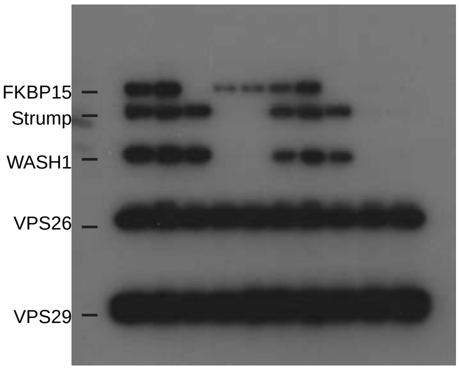

WB: FKBP15, Strumpellin, WASH1, VPS26, VPS29
(higher exp)

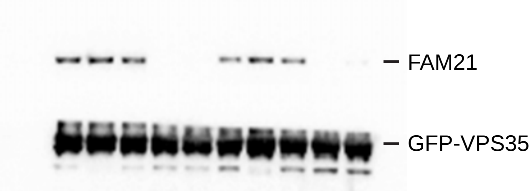

WB: FAM21, VPS35
(phosphoimager image)

Figure 3d

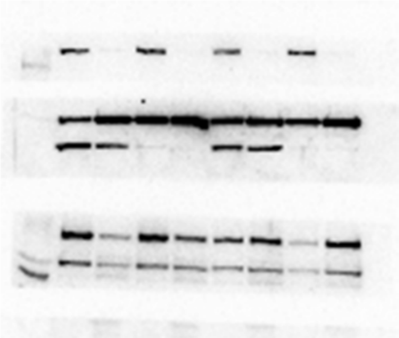

Supplementary Figure 12. continued

Figure 4a

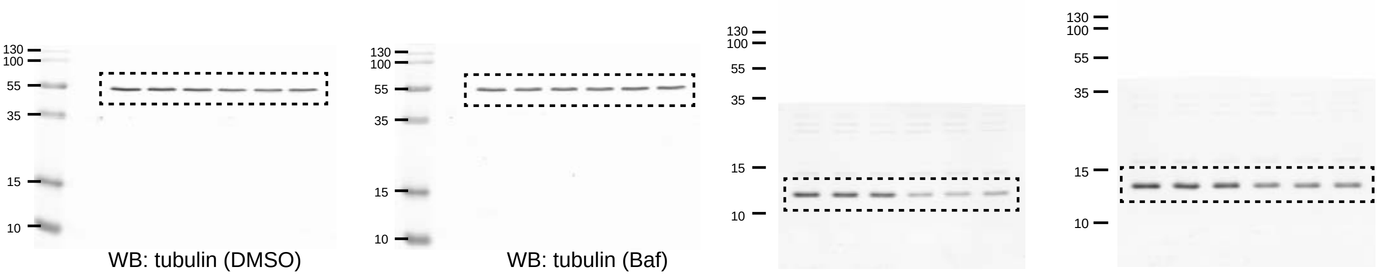

WB: tubulin (DMSO) WB: tubulin (Baf) WB: LC3 (DMSO) WB: LC3 (Baf)

Figure 4f

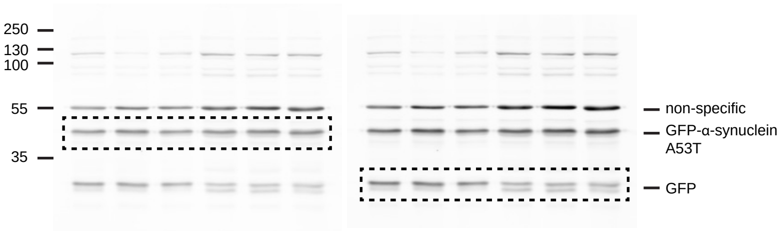

WB: GFP (lower exp) WB: GFP (higher exp)

Figure 4h

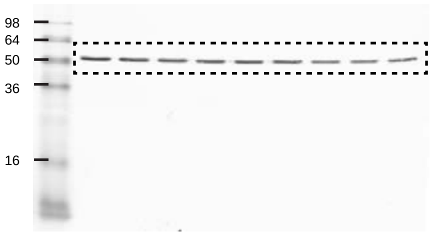

WB: tubulin (DMSO)

Figure 4h, cont

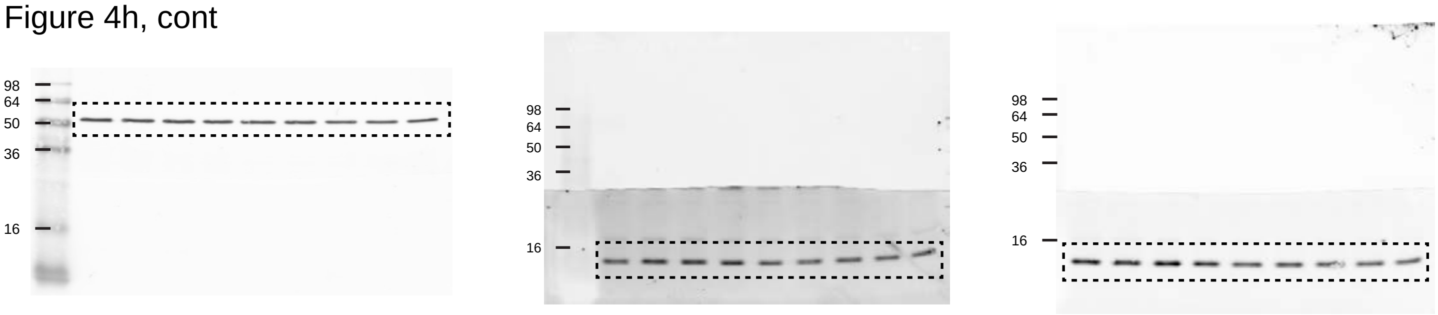

WB: tubulin (Baf) WB: LC3 (DMSO) WB: LC3 (Baf)

Figure 4j

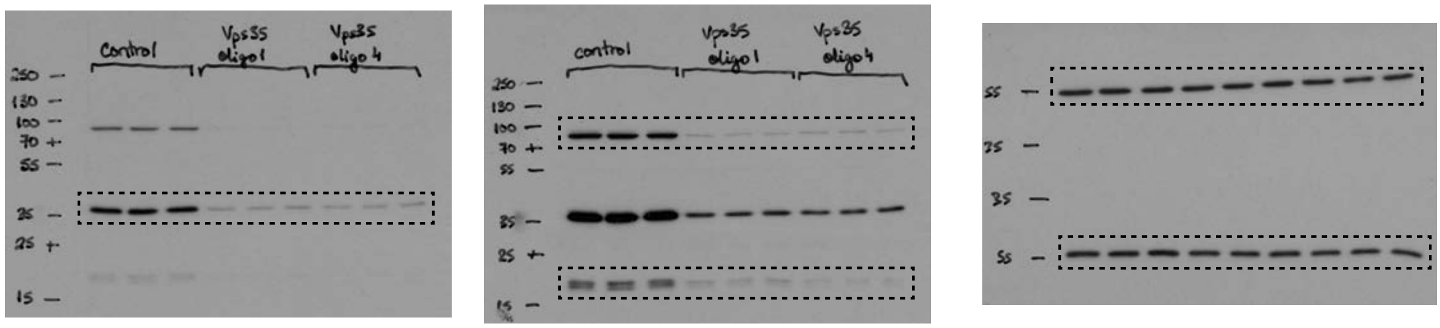

WB: VPS35, VPS26, VPS29
(DMSO, lower exp.)

WB: VPS35, VPS26, VPS29
(DMSO, higher exp.)

WB: tubulin
(DMSO - top, Baf - bottom, shown upside down)

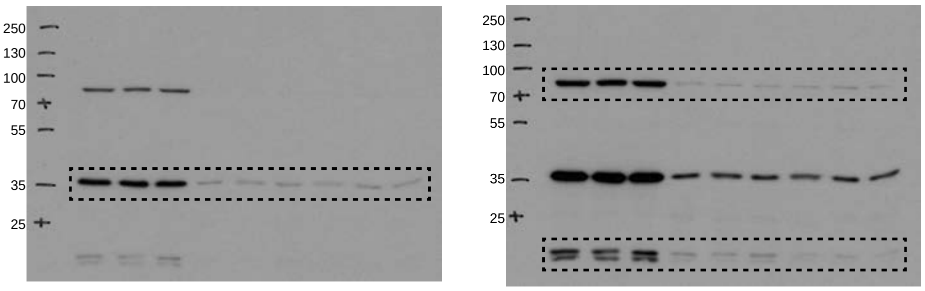

WB: VPS35, VPS26, VPS29
(Baf, lower exp.)

WB: VPS35, VPS26, VPS29
(Baf, higher exp.)

Supplementary Figure 12. continued

Figure 5a

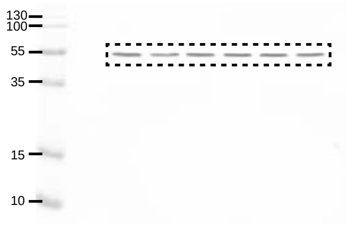

WB: tubulin (DMSO)

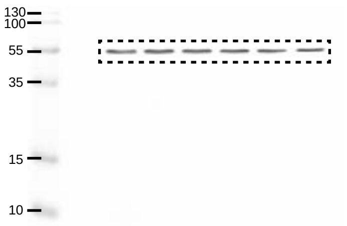

WB: tubulin (Baf)

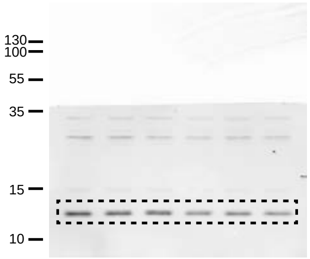

WB: LC3 (DMSO)

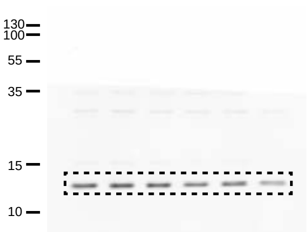

WB: LC3 (Baf)

Figure 5c

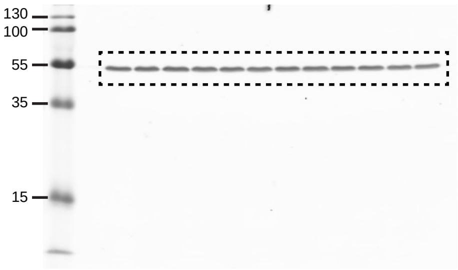

WB: tubulin

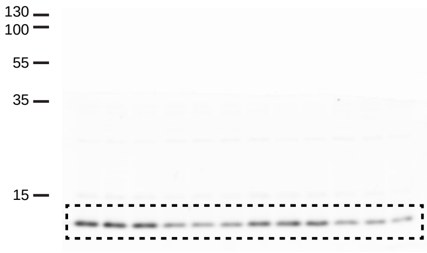

WB: LC3 (lower exp)

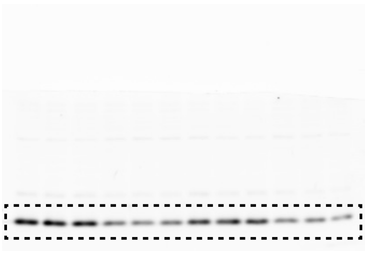

WB: LC3 (higher exp)

Figure 5e

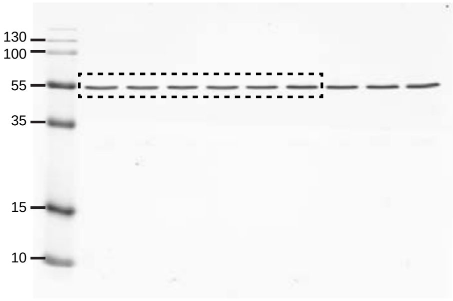

WB: tubulin (DMSO)

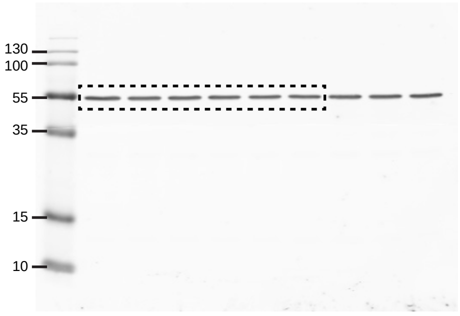

WB: tubulin (Baf)

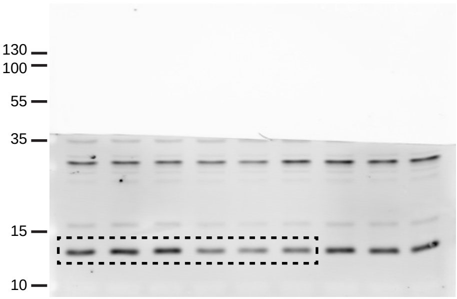

WB: LC3 (DMSO)

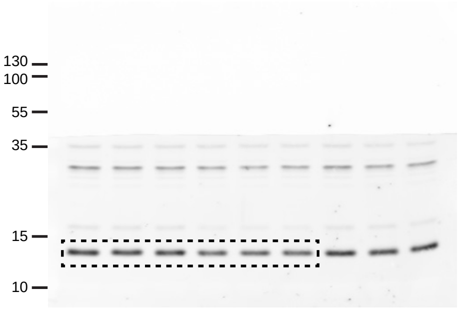

WB: LC3 (Baf)

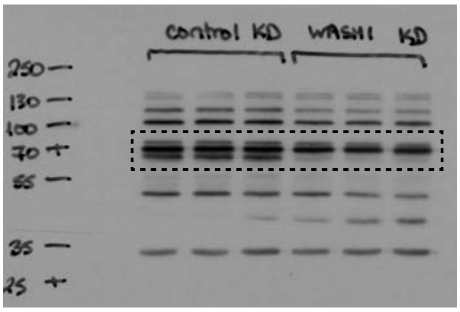

WB: WASH1 & GAPDH (DMSO, lower exp)

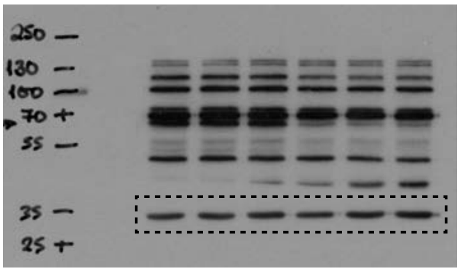

WB: WASH1 & GAPDH (DMSO, higher exp)

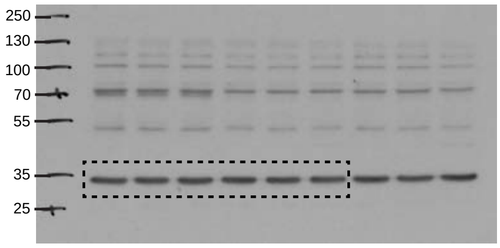

WB: WASH1 & GAPDH (Baf, lower exp)

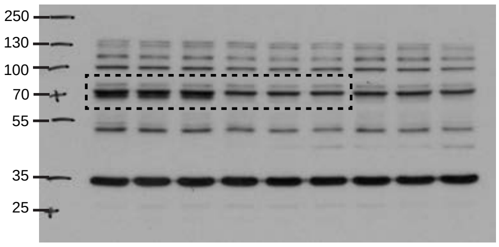

WB: WASH1 & GAPDH (Baf, higher exp)

Supplementary Figure 12. continued

Figure 5j

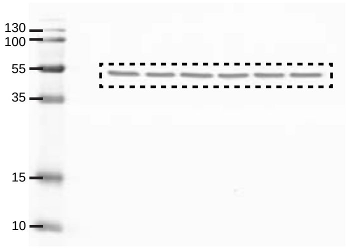
WB: tubulin (DMSO)

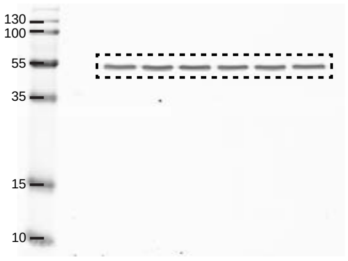
WB: tubulin (Baf)

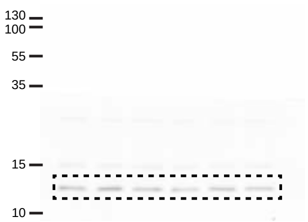
WB: LC3 (DMSO, lower exp)

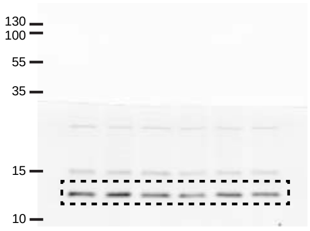
WB: LC3 (DMSO, higher exp)

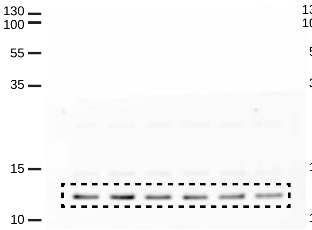
WB: LC3 (Baf, lower exp)

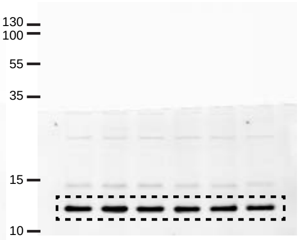
WB: LC3 (Baf, higher exp)

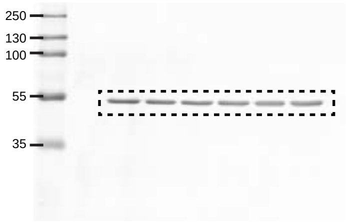
WB: tubulin (DMSO)

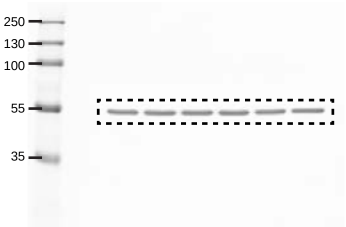
WB: tubulin (Baf)

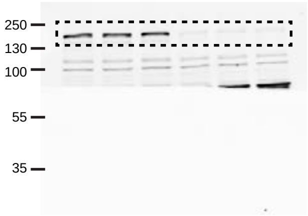
WB: FKBP15 (DMSO)

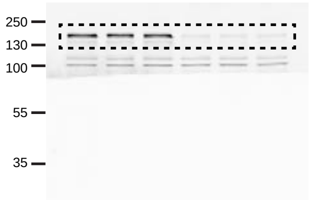
WB: FKBP15 (Baf)

Figure 9a

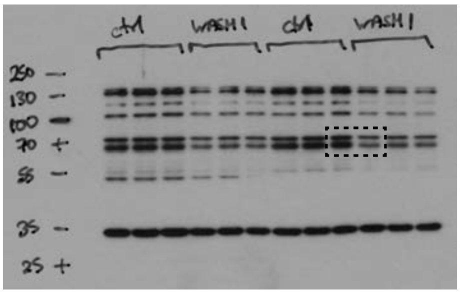
WB: WASH1, GAPDH (high exp)

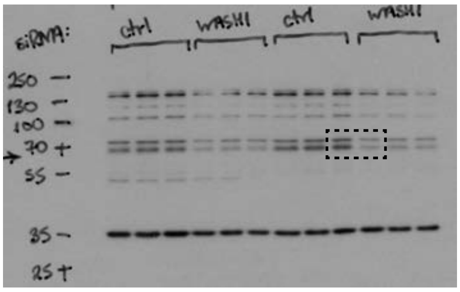
WB: WASH1, GAPDH (medium exp)

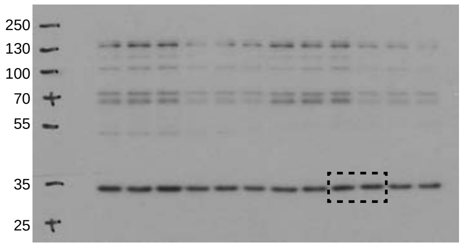
WB: WASH1, GAPDH (low exp)

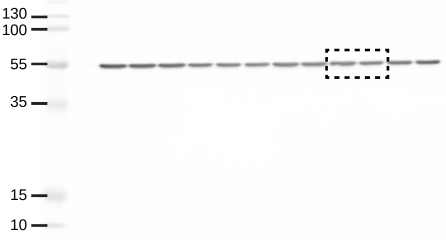
WB: tubulin

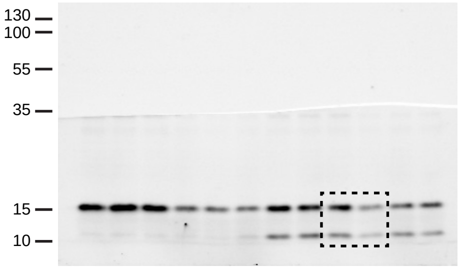
WB: LC3

Supplementary Figure 12. continued

Supplementary Figure 1a

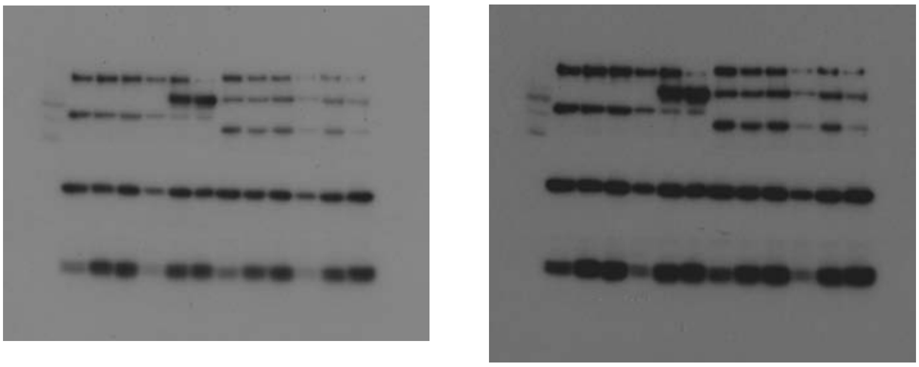

WB: FAM21, Strumpellin, WASH1, FKBP15, VPS35, VPS26, VPS29
(lower exp)

WB: FAM21, Strumpellin, WASH1, FKBP15, VPS35, VPS26, VPS29
(higher exp)

Supplementary Figure 1b

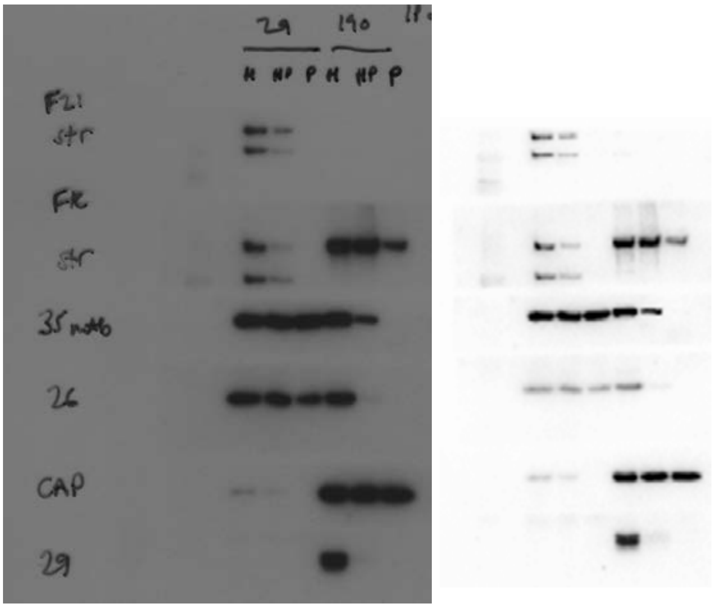

WB: FAM21, Strumpellin, VPS35, VPS26, CAPZa, VPS29
(right image is from phosphoimager)

Supplementary Figure 1c

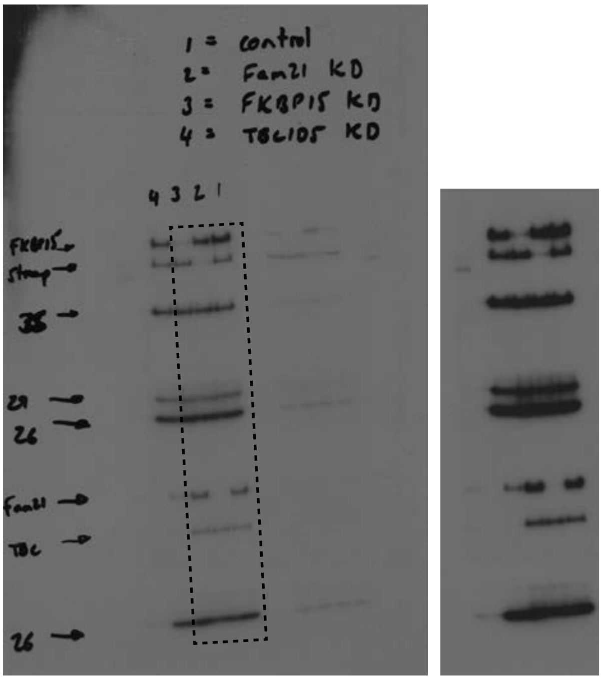

WB: FKBP15, Strumpellin, VPS35, VPS29, VPS26, FAM21, TBC1D5
(lower exp)

(higher exp)

Supplementary Figure 1d

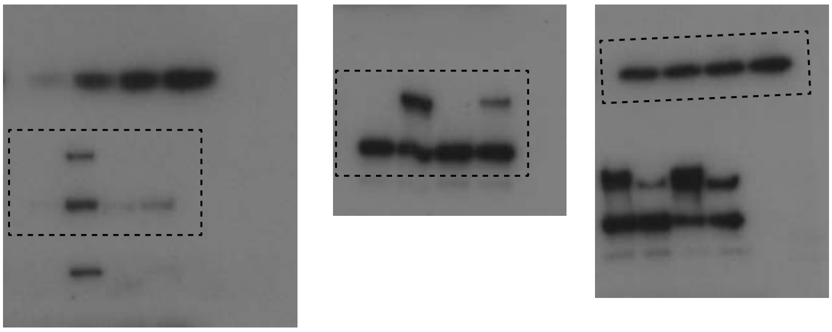

WB: FAM21, Strumpellin

WB: FKBP15, VPS35

WB: VPS26

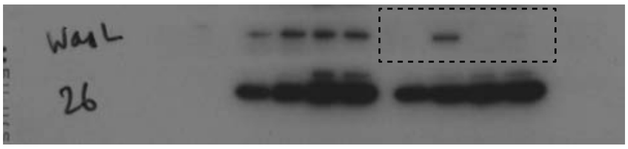

WB: WASH1, VPS26

Supplementary Figure 12. continued

Supplementary Figure 4a

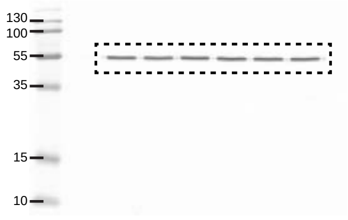

WB: tubulin (DMSO)

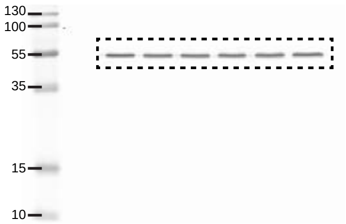

WB: tubulin (Baf)

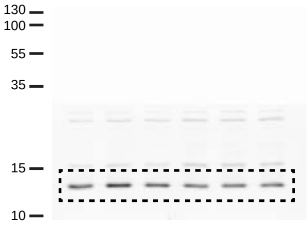

WB: LC3 (DMSO)

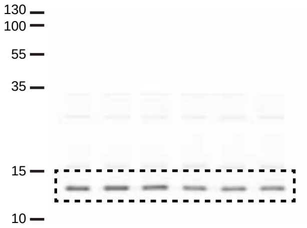

WB: LC3 (Baf)

Supplementary Figure 4c

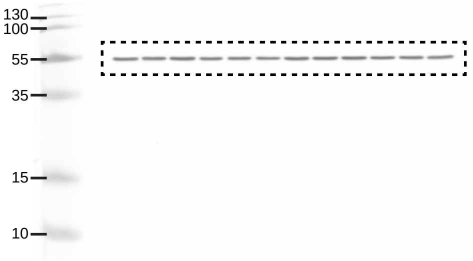

WB: tubulin (DMSO)

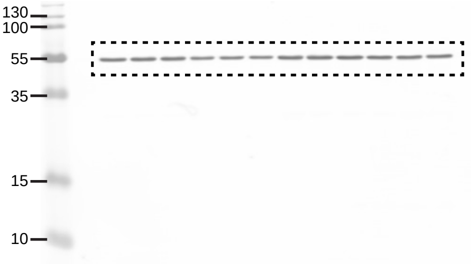

WB: tubulin (Baf)

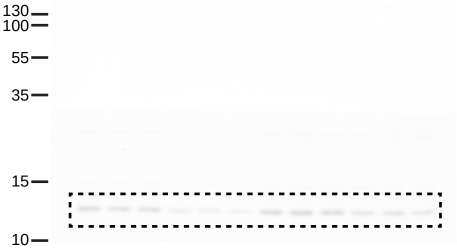

WB: LC3 (DMSO, lower exp)

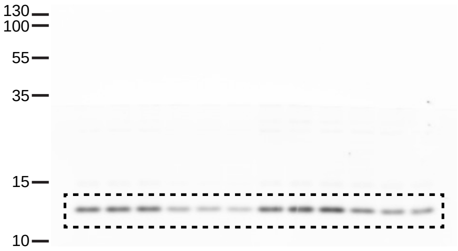

WB: LC3 (Baf, lower exp)

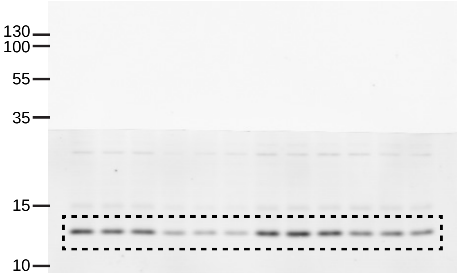

WB: LC3 (DMSO, higher exp)

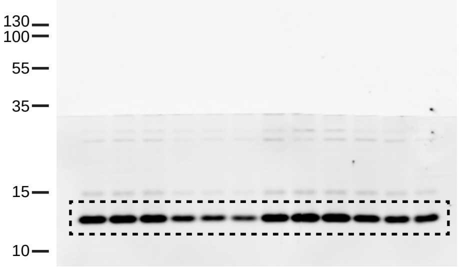

WB: LC3 (Baf, higher exp)

Supplementary Figure 4e

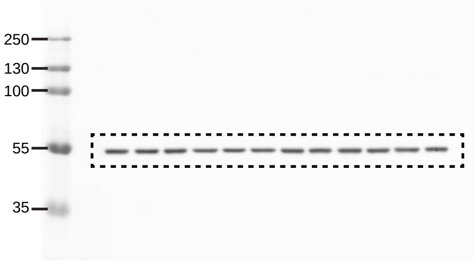

WB: tubulin (DMSO)

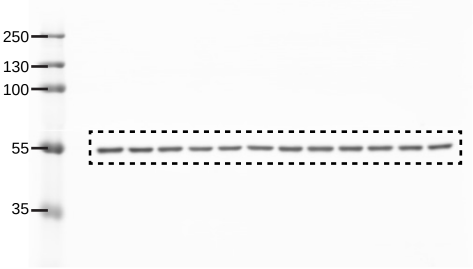

WB: tubulin (Baf)

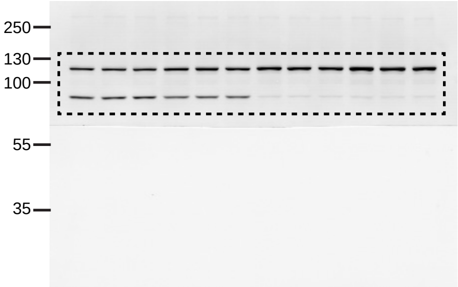

WB: VPS35 (DMSO)

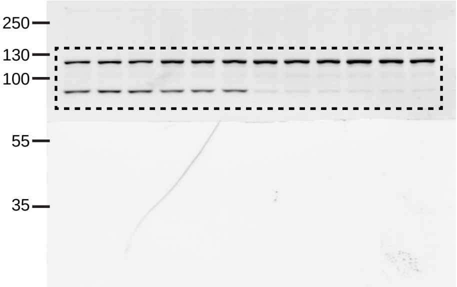

WB: VPS35 (Baf)

Supplementary Figure 12. continued
